# Cellular Growth Arrest and Efflux Pumps Are Associated With Antibiotic Persisters in *Streptococcus pyogenes* Induced in Biofilm-Like Environments

**DOI:** 10.3389/fmicb.2021.716628

**Published:** 2021-09-21

**Authors:** Caroline Lopes Martini, Amada Zambrana Coronado, Maria Celeste Nunes Melo, Clarice Neffa Gobbi, Úrsula Santos Lopez, Marcos Correa de Mattos, Thais Tavares Amorim, Ana Maria Nunes Botelho, Ana Tereza Ribeiro Vasconcelos, Luiz Gonzaga Paula Almeida, Paul J. Planet, Russolina Benedeta Zingali, Agnes Marie Sá Figueiredo, Bernadete Teixeira Ferreira-Carvalho

**Affiliations:** ^1^Instituto de Microbiologia Paulo de Góes, Universidade Federal do Rio de Janeiro, Rio de Janeiro, Brazil; ^2^Departamento de Microbiologia e Parasitologia, Universidade Federal do Rio Grande do Norte, Natal, Brazil; ^3^Laboratório Nacional de Computação Científica (LNCC), Petrópolis, Brazil; ^4^Department of Pediatrics, Perelman College of Medicine, University of Pennsylvania, Philadelphia, PA, United States; ^5^Sackler Institute for Comparative Genomics, American Museum of Natural History, New York, NY, United States; ^6^Children’s Hospital of Philadelphia, Philadelphia, PA, United States; ^7^Unidade de Espectrometria de Massas e Proteomica – UEMP, Instituto de Bioquímica Médica Leopoldo de Meis, Universidade Federal do Rio de Janeiro, Rio de Janeiro, Brazil

**Keywords:** *Streptococcus pyogenes*, drug refractory, persisters, efflux pump, antimicrobial resistance, clinical resistance

## Abstract

*Streptococcus pyogenes* (group A *Streptococcus*-GAS) is an important pathogen for humans. GAS has been associated with severe and invasive diseases. Despite the fact that these bacteria remain universally susceptible to penicillin, therapeutic failures have been reported in some GAS infections. Many hypotheses have been proposed to explain these antibiotic-unresponsive infections; however, none of them have fully elucidated this phenomenon. In this study, we show that GAS strains have the ability to form antimicrobial persisters when inoculated on abiotic surfaces to form a film of bacterial agglomerates (biofilm-like environment). Our data suggest that efflux pumps were possibly involved in this phenomenon. In fact, gene expression assays by real-time qRT-PCR showed upregulation of some genes associated with efflux pumps in persisters arising in the presence of penicillin. Phenotypic reversion assay and whole-genome sequencing indicated that this event was due to non-inherited resistance mechanisms. The persister cells showed downregulation of genes associated with protein biosynthesis and cell growth, as demonstrated by gene expression assays. Moreover, the proteomic analysis revealed that susceptible cells express higher levels of ribosome proteins. It is remarkable that previous studies have reported the recovery of *S. pyogenes* viable cells from tissue biopsies of patients presented with GAS invasive infections and submitted to therapy with antibiotics. The persistence phenomenon described herein brings new insights into the origin of therapeutic failures in *S. pyogenes* infections. Multifactorial mechanisms involving protein synthesis inhibition, cell growth impairment and efflux pumps seem to play roles in the formation of antimicrobial persisters in *S. pyogenes*.

## Introduction

Group A streptococci (GAS) has long been recognized as one of the most important disease-causing bacteria in humans. These bacteria are associated with different types of infections, including pharyngitis, impetigo, scarlet fever, cellulitis and abscesses. GAS is also involved in severe invasive infections such as myositis and necrotizing fasciitis, and cases of toxic shock syndrome. Additionally, some post infectious sequelae have been reported (Avire et al., 2021).

GAS strains are typically susceptible to penicillin (Oppegaard et al., 2020). However, studies have reported treatment failures of patients receiving β-lactam therapy (Gidengil et al., 2013; Brook, 2017; Randhawa et al., 2018). Many explanations have been proposed to elucidate clinical failures of penicillin treatment, including protection of GAS-susceptible isolates by β-lactamase producers in the pharyngeal microbiota, penicillin tolerance, biofilm formation, bacterial internalization in host cells among others (Thulin et al., 2006; Walker et al., 2014; Fiedler et al., 2015; Brook, 2017). However, the contribution of each of these mechanisms for drug failures remains unclear (Walker et al., 2014; Brook, 2017).

Several mechanisms have also been described in other bacterial species to explain phenotypic drug resistance including tolerance, small colony variants (SVCs), heteroresistance and persisters (Brauner et al., 2016; Balaban et al., 2019; Proctor, 2019; Yu et al., 2019; Lee et al., 2020). The phenomenon of tolerance is defined for bactericidal antibiotics when the minimum inhibitory concentration (MIC) of the tolerant is equal to that of the susceptible strain, but the minimal bactericidal concentration (MBC) and the time required for bacterial death to occur are considerably higher (Brauner et al., 2016; Balaban et al., 2019). SCVs are characterized by their slow growth resulting in small colony sizes, which show mutations in genes often associated with auxotrophic phenotypes, electron transportation chain, and biosynthetic pathways (Proctor, 2019; Lee et al., 2020). Heteroresistance defines a mechanism by which cell subpopulations in a bacterial culture are killed by different antibiotic concentrations. Therefore, although most cells are eliminated at the MIC value, a few can survive. Nevertheless, they are often killed at certain drug concentration not far from MIC, leading to low-level (borderline) resistance. Additionally, heteroresistance is generally defined for an antimicrobial class while persisters are often resistant to different classes and remain viable in antimicrobial concentrations far above the MIC (Balaban et al., 2019). In the mechanism of persister generation, fraction of the bacterial population switches stochastically to the persister phenotype during the growth phase. An important characteristic of the persisters is the occurrence of slow- or non-growing bacterial cells that remain viable during exposure to antibiotics. Despite that, antibiotic susceptibility is regained after bacterial growth in the absence of the drug (Pontes and Groisman, 2019; Yu et al., 2019; Huemer et al., 2020). Therefore, the demonstration of growth impairment in the presence of antibiotics is important before they can be classified as persisters (Yu et al., 2019; Pontes and Groisman, 2019).

In the study herein we report the generation of antimicrobial-persisters by GAS cells in a biofilm-like environment and investigate some mechanisms known to be associated with persisters in other bacterial pathogens. The formation of GAS persisters may be also a mechanism behind antimicrobial failures that has been overlooked.

## Materials and Methods

### Bacterial Isolates

Two hundred-eleven GAS isolates were used to test the emergence of persister cells to β-lactam antibiotics. These isolates belong to a convenience collection obtained from infected patients and colonized individuals, in different Brazilian cities, from different clinical sites (Supplementary Table 1). The majority of these isolates were from outpatient cases of symptomatic oropharyngeal infections, and obtained from 1978 to 1997. Clonality were previously analyzed by pulsed-field gel electrophoresis (PFGE) for roughly half of these isolates, which displayed extensive genetic diversity (Melo et al., 2003). These GAS were identified by routine methods and confirmed by latex agglutination tests (Streptococcal Grouping Kit; Oxoid, Basingstoke, Hampshire, United Kingdom). Minimal inhibitory concentration (MIC) for all antimicrobials used in this study, except azithromycin and ethidium bromide (EtBr), was previously determined for this GAS collection (Melo et al., 2003). Since all GAS isolates analyzed were equally able to produce persisters under the experimental model used, to get some insights into the molecular mechanisms associated with antimicrobial persisters in GAS we randomly choose the GAS strain 37–97 among the isolates of this collection whose PFGE patterns were previously determined. This strain showed sequence type (ST) 62, *emm* 87 and was isolated from symptomatic oropharynx infection case, in 1997, in the outpatient clinic of the Hospital de Puericultura Martagão Gesteira, Rio de Janeiro, RJ. Additionally, nine other GAS isolates were chosen from the convenience collection based on diverse PFGE patterns, different clinical sources, and susceptibility to all antimicrobials tested (Supplementary Table 2). These nine isolates were used as control in the phenotypic tests to detect persisters to validate the results obtained for the representative strain 37–97. Pure cultures of the 211 GAS isolates analyzed were obtained from lyophilized stocks. One tube of each isolate was opened and after reconstitution, cultures was stored at −80°C in brain heart infusion (w/v) with 0.5% (w/v) of yeast extract and 18% (v/v) glycerol.

### Minimal Inhibitory Concentration

MIC determinations for azithromycin (Azi; Sigma, St. Louis, MO, United States) and ethidium bromide (EtBr; Sigma) were done using the agar dilution method as recommended by the Clinical & Laboratory Standards Institute (CLSI, 2021) with concentrations ranging from 0.06 to 4 μg/mL and 0.015 to 4 μg/mL; respectively. Two biological experiments were performed (*N* = 2).

### Development of GAS-Persister Cells to β-Lactams

The model used in this study to generate persisters was based on previous work done with *Staphylococcus aureus* strains (Novais et al., 2020). In this system (here called biofilm like-environment) high bacterial load is inoculated in order to allow the formation of an initial bacterial film on the smooth surface of a cellophane membrane placed onto agar media containing antibiotics to mimic bacterial agglomeration found in some environments such as those encountered in biofilms. Persisters were indirectly detected in the system containing antibiotic by CFU counting (Orman and Brynildsen, 2015; Yu et al., 2019). To prepare the bacterial inoculum, GAS isolates (*n* = 211) were grown in Todd Hewitt broth containing 0.5% (w/v) of yeast extract (THB-Y) at 37°C/6 h in order to reach the exponential phase. After centrifugation, the pellet was adjusted (∼ 1–2 × 10^10^ colony forming unit-CFU/mL) using the same broth. To form a bacterial film, a 100-μL volume (∼ 2–4 × 10^7^ CFU/cm^2^) was homogeneously spread on the surface of a cellophane membrane placed onto THB-Y agar containing 5% defibrinated sheep blood (BAB) and supplemented with 0.005–8 μg/mL penicillin (Pen; Wyeth-Whitehall Ltda, Itapevi, SP, Brazil) or 0.25–4 μg/mL cephalexin (Cep; Sigma). After 37°C/18 h, persisters were removed from the cellophane membranes at the highest drug concentration in which growth was detected for CFU counting. To test whether defibrinated sheep blood interfered with the analysis, the experiments were also performed in the absence of blood. Antimicrobial susceptible control cells were obtained exactly as described above but using inoculum size adjusted to concentrations recommended by CLSI (∼10^6^CFU/plate; condition that does not allow the generation of persisters). Four biological experiments were performed with two technical replicates each. CFU determinations were carried out for the representative strain 37-97. Two CFU determinations were carried out for each dilution (*N* = 4).

### Proteomic Analysis

A proteomic analysis was done to assess protein differential expression between cells grown in biofilm-like environment (condition that allows generation of persisters) and GAS susceptible cells (inoculum size adjusted to ∼10^6s^CFU/plate, condition that does not promote antibiotic persistence). Bacterial cells from strain 37–97 were collected from the cellophane membrane, suspended in phosphate buffered saline (PBS) (140 mM NaCl; 2.7 mM KCl; 8 mM Na_2_HPO_4_, and KH_2_PO_4_ 1.5 mM; pH 7.2) using vigorous shaking, and adjusted to OD_600 nm_ = 0.4. Pellet was washed twice, resuspended in PBS and lysed with 106 μm beads (Sigma) in a Bio101 Fast Prep system (BioSavant, Qbiogene, Carslbad, CA, United States) using six cycles (5 speeds/30 s pulse). After centrifugation, the protein concentration was estimated using a Qubit 2.0 (Invitrogen Life Technologies, CA, United States), and lysates diluted in sodium dodecyl sulfate polyacrylamide Gel (SDS-PAGE) sample buffer (1:1, v/v) (Laemmli, 1970). Proteins were separated using a 12.5% SDS-PAGE gel electrophoresis, and individual bands were isolated from the gels. All procedures used for the treatment of gel slices and trypsin digestion were performed as previously described (Shevchenko et al., 1996). The resulting peptides were desalted using an in-house reverse-phase microcolumn (POROS R2 resin, Applied Biosystems, Carlsbad, CA, United States) and dried by vacuum centrifugation (Rodrigues et al., 2011). Peptides were solubilized in 20 μL of 0.1% (v/v) formic acid (FA), and 10 μL were injected into a trap column (Opti-Pak C18, Waters, Milford, MA, United States). Liquid chromatography separation was performed using a reverse-phase capillary column (nanoEase C18, 100 mm × 100 μm, Waters) connected to a nano-HPLC system (Waters UPLC, Waters). The eluted peptides were introduced into an ESI-Q-TOF-MS/MS (Q-TOF Micro, Waters) controlled by MassLynx software (Version 4.1, Waters). Mass spectra (MS) were collected in the 50–2,000 m/z range, and the three most abundant ions (charges +2, +3, and +4) were submitted for collision-induced dissociation (CID) using argon gas at 13 psi and 18–45 V. The raw data were converted to a peak list using the ProteinLynx Global software (version 4.0, Waters). Protein identification was considered valid if at least one peptide with minimum of 10 amino acids was observed with a maximum error tolerance of 50 ppm and Mascot score ≥ 46 (*p* ≤ 0.05). The GenBank (Acc) access number, locus tag, and gene and protein names were determined using BLASTp.^1^ In addition, Uniprot BLAST analysis^2^ was performed in order to identify homologs in *S. pyogenes* MGAS10750. Only *e*-values ≤ 1.0 e–3 were considered in the database search.

### Detection of Ethidium Bromide-Refractory Cells

The increase in ethidium bromide (EtBr) MIC values is highly sensitive and specific in identifying efflux-proficient strains in *S. aureus* (Patel et al., 2010). Therefore, we evaluated the occurrence of EtBr-refractory cells in the biofilm-like environment. Different EtBr concentrations (0.015–4.0 μg/mL) were added to BAB agar that was covered with cellophane membranes. High-bacterial load was placed onto the surface of cellophane membranes to produce a biofilm-like environment as described before. After 18 h incubation (37°C), GAS cells were recovered from the cellophane membranes at the highest EtBr concentration in which growth was detected for CFU determinations. Controls were performed exactly as above but with susceptible cells (∼10^6^ CFU/plate). Four biological experiments were performed with two technical replicates each. CFU determinations were carried out for the representative strain 37–97. Two CFU determinations were carried out for each dilution (*N* = 4).

### Persistence to Non-β-Lactam Antibiotics

These experiments were performed to assess if the persistence observed for GAS cells was associate to β-lactams only, or to universal mechanisms as those involving efflux pumps. The ability of GAS strain 37–97 to become refractory to different antimicrobials was tested in concentrations ranging from 0.01 to 4 μg/mL erythromycin (Ery; Sigma), 0.06–4 μg/mL azithromycin (Azi; Sigma), 0.01–1 μg/mL clindamycin (Cli; Sigma), 0.25–16 μg/mL chloramphenicol (Chl; Sigma), or 0.125–16 μg/mL tetracycline (Tet; Sigma). Plates were examined after incubation for 18 h at 37°C. Controls were also performed exactly as above but with susceptible cells (∼10^6^ CFU/plate). Additionally, for control purposes, these experiments were also done with additional nine GAS isolates (Supplementary Table 2), using the highest concentration of antibiotic in which bacterial growth was detected for the representative strain 37–97. For each antimicrobial tested, two to six biological experiments were performed with two technical replicates each. CFU determinations were carried out for the representative strain 37–97. Two CFU determinations were carried out for each dilution. Ery (*N* = 2), Azi (*N* = 4), Cli (*N* = 6), Chl (*N* = 6), Tet (*N* = 6).

### Effect of Cyanide 3-Chlorophenylhydrazone (CCCP) Efflux Pump Inhibitor in Antimicrobial Refractory

Because CCCP inhibits proton motive force and also the transcription of some transport associated genes (Baron and Rolain, 2018), the ability of this compound to inhibit the formation of persister cells was tested. The CCCP (Sigma) at 100 μM final concentration (Clancy et al., 1996) was incorporated to BAB (covered or not with a cellophane membrane) supplemented with 8 μg/mL penicillin (MIC = 0.01 μg/mL), 4 μg/mL cephalexin (MIC = 0.5), 4 μg/mL erythromycin (MIC = 0.12 μg/mL), 4 μg/mL azithromycin (MIC = 0.12 μg/mL), 1 μg/mL clindamycin (MIC = 0.01 μg/mL), 16 μg/mL chloramphenicol (MIC = 1.0 μg/mL), 16 μg/mL tetracycline (MIC = 0.12 μg/mL), or 4 μg/mL EtBr (MIC = 0.06 μg/mL). BAB plates without CCCP were used to control bacterial growth. High bacterial load was placed onto the surface of cellophane membranes or uncovered BAB plates. After 18 h incubation (37°C), all plates were examined and GAS cells recovered from the cellophane membranes at the highest drug concentration in which growth was detected for CFU determinations. Controls were performed exactly as above but with susceptible cells (∼10^6^ CFU/plate). Three biological experiments were performed with two technical replicates each for the representative strain 37–97. Two CFU determinations were carried out for each dilution (*N* = 3).

### Phenotypic Switching Test

GAS persister cells of the strain 37–97 recovered from the cellophane membranes covering BAB plates with 8 μg/mL penicillin were subjected to successive passages (up to 500 generations) on BAB without antibiotics. After passaging, bacterial growth was adjusted to concentrations recommended by CLSI (∼10^6^ CFU/plate), and the penicillin MIC was determined using the agar dilution method (CLSI, 2021). Two biological experiments were performed (*N* = 2).

### Whole-Genome Sequencing

For total DNA preparation, penicillin-persister (8 μg/mL penicillin plates; MIC 0.01 μg/mL) and -susceptible cells of the strain 37–97 were recovered from cellophane membranes. An aliquot of the cell suspension was inoculated in THB-Y (1:200 dilution). After incubation (37°C/18 h), DNA was obtained using the Wizard Genomic DNA Purification Kit (Promega; Madison, WI, United States). Genomic libraries were prepared using the Nextera XT kit (Illumina, San Diego, CA, United States) and sequenced on an Illumina HiSeq (125 pb reads). Reads were trimmed using BBDuk Trimmer (version 1.0) and genome assembly was carried out using Newber v3.0 (Margulies et al., 2005). Scaffolds were aligned against a reference genome (*S. pyogenes* strain NGAS743; Acc: CP007560) using cross match (version 0.990329).^3^ Intra-scaffold and inter-scaffold gaps resulting from repetitive sequences were resolved by *in silico* gap filling. Any remaining gaps in the genomic sequence from penicillin-persister cells of the 37–97 strains (37–97P) were filled with “N” with estimated sizes based on the complete sequence of the susceptible cells of the strain 37–97 (37–97S). The sequenced genomes were annotated using RAST 2.0v (Overbeek et al., 2014). Taxonomic analysis was performed by calculating average nucleotide identity (ANI) for whole genomes using OrthoANIu tool.^4^ Multi locus sequence typing (MLST) was performed for the genome sequences using MLST 2.4.0 software.^5^ Differences in single nucleotide polymorphisms (SNPs) between samples 37–97S and 37–97P were evaluated using cross match with parameter discrep lists. The generated list was compared to the Newbler assembly ace file and genome annotation. SNPs were verified by resequencing on an ABI 3730 DNA Analyzer (Life Technologies—Applied Biosystem; Carlsbad, CA, United States). Reactions were performed using the BigDye Terminator v3.1 Cycle Sequencing Kit in 36-cm capillaries with POP7 polymer according to the manufacturer’s instructions.

### Gene Expression Analysis

Total RNA from penicillin-persisters and -susceptible cells obtained from 37–97 strain was prepared from a suspension of cells directly recovered from cellophane membranes as described in the item “Development of GAS-persister cells to β-lactams.” The RNeasy Mini kit (Qiagen; Germantown, MD, United States) was used for RNA preparation that was quantified by a Qubit 2.0 Fluorometer (Thermo Fisher Scientific Brasil; São Paulo, SP, Brazil). RNA quality was analyzed by gel electrophoresis. For some experiments, gene expression was also performed in presence of 100 μM CCCP. To test the effect of clindamycin in the expression of the efflux-associated locus *MGAS10750_Spy1819*, total RNA was prepared from GAS persister cells recovered from cellophane membranes on BAB plates containing 1 μg/mL clindamycin (MIC = 0.01 μg/mL). The real-time quantitative reverse transcriptase PCR (real-time RT-qPCR) was performed using Power SYBR Green RNA-to-CT^TM^ 1-Step Kit (Applied Biosystems) as recommended (“Guide to Performing Relative Quantitation of Gene Expression Using Real-Time Quantitative PCR”; Applied Biosystems). The rRNA 16S gene was used as an endogenous control. The calibrator sample was total RNA from susceptible cells of strain 37–97. The reaction was performed in a Step One^TM^ Real Time PCR System (Applied Biosystems). Data were analyzed using Step One Software 2.2 (Applied Biosystems). All primers were validated as recommended in the cited guide and listed in Supplementary Table 3. Three biological replicates were performed with three technical replicates each (*N* = 3).

### Statistical Tests

To analyze the quantity of persister cells recovering in presence of β-lactams and other antimicrobial classes, one way ANOVA was applied followed by *post hoc* Tukey’s test for multiple comparisons. Two-tailed unpaired Student’s *t*-test was used to analyze most the binary experiments of gene expression. To analyze the hypothesis that the expression of efflux pump-associated genes increases in the persister GAS cells, one-tailed unpaired *t*-test was performed. All statistical tests were calculated using GraphPad Prism version 9.2.0 for Windows (GraphPad Software, La Jolla, CA, United States). In addition, to confront the null hypothesis, Scaled Jeffreys–Zellner–Siow (JZS) Bayes Factor for two-samples *t*-test was calculated to test the alternative hypothesis for *r* = 0.707 (Rouder et al., 2009).

## Results

### Persistence to β-Lactam Antibiotics

**FIGURE 1:**
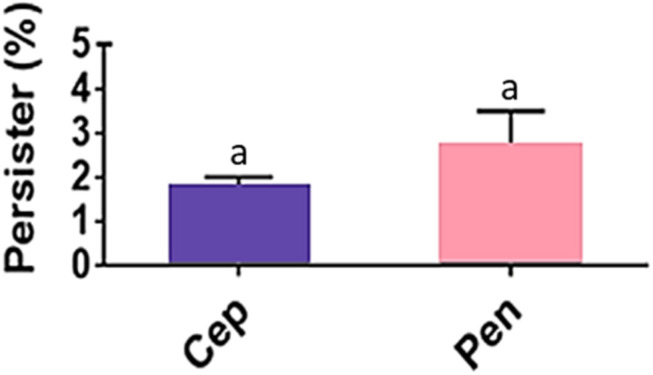
*Streptococcus pyogenes* persisters recovered from biofilm-like environments at concentrations of 4 μg/mL cephalexin (Cep; MIC = 0.5 μg/mL) or 8 μg/mL penicillin (Pen; MIC = 0.01 μg/mL). The average CFU/mL of the control cells (no antibiotic) was 6.0 × 10^10^ and corresponded to 100%. One way ANOVA (*p* < 0.001; *F* = 16.81; DF_to__tal_ = 11) was applied for CFU values. *Post hoc* Tukey’s test was performed followed ANOVA for multiple comparisons between the control and antibiotics (^*a*^*p* < 0.01) and between antibiotics (there was no significant difference in the amount of persister cells recovered when Cep and Pen were compared; *p* = 0.9712).

Despite the susceptible MIC values for penicillin (MIC range = 0.0025–0.02 μg/mL; MIC50 and MIC90 = 0.01 μg/mL), persisters were detected for the 211 GAS isolates in all penicillin concentrations tested including those far above MIC and as high as 8 μg/mL. To observe a possible influence of defibrinated sheep blood on the formation of persisters, GAS strain 37–97 was inoculated on BAB plates with and without blood supplementation, both containing 8 μg/mL penicillin, covered or not with cellophane membranes. Persister cells were equally formed when high bacterial load was inoculated. The average detection of persister corresponded to 2.7% (*p* = 0.0022) of the total cell population grown in the absence of penicillin (6.0 ± 2.4 × 10^10^ CFU/mL) (Figure 1). When uncovered BAB plates were examined, persisters formed almost invisible (very tiny) hemolytic colonies, which returned to the normal size after passage in fresh media without antibiotics. Similar to the results obtained for penicillin, persisters could also arise on BAB plates containing 4 μg/mL cephalexin (MIC50 and MIC90 = 0.5 μg/mL). The mean percentage of persisters for 4 μg/mL cephalexin was 1.8% (*p* = 0.0016) of the total cell population grown in the absence of the drug (6.0 ± 2.4 × 10^10^ CFU/mL) (Figure 1).

Drug susceptibility could be reverted when persister cells were submitted to serial passaging on BAB plates without penicillin, with the antibiotic persistent cells returning to their original state of drug susceptibility (MIC = 0.01 μg/mL). To assess whether this persistence phenotype was actually induced by the biofilm-like environment or due to preexistent heterogeneous resistant subpopulations present in the high bacterial load provided by the heavy inoculum size (∼ 1–2 × 10^9^ CFU/100 μL), this inoculum was divided in 100 parts. To each part, 99.9 μL of THB-Y was added and the total 100 μL inoculated onto a cellophane membrane on the BAB plate containing 8 μg/mL penicillin. To control this experiment, the total inoculum (∼ 1–2 × 10^9^ CFU/100 μL) was also inoculated onto a cellophane membrane on the BAB plate with 8 μg/mL penicillin. After 18 h at 37°C, persisters were only generated in the environment of cell agglomeration of the control. No growth was detected in the 100 plates inoculated with part of the inoculum, clearly ruling out the presence of heteroresistant subpopulations in the GAS culture.

**FIGURE 2:**
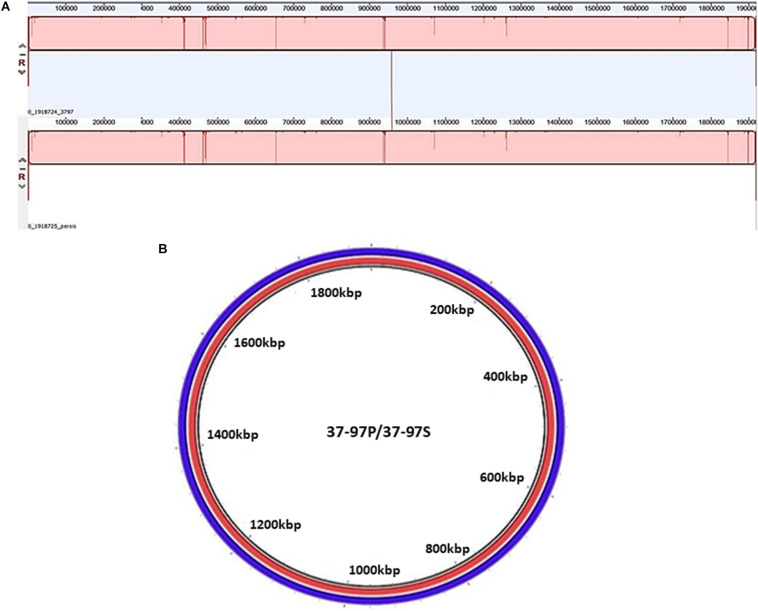
Analysis of synteny and circular comparison of the chromosomes. **(A)** Genome alignments were performed using MAUVE. **37–97P**: DNA sequence of the penicillin persister cells of the *Streptococcus pyogenes* strain 37-97 generated in 8 μg/mL penicillin (MIC = 0.01 μg/mL), and **37–97S**: DNA sequence of the susceptible cells of strain 37–97. **(B)**. Circular comparison of the chromosomes was generated by Blast Ring Image Generator (BRIG) using genome sequences obtained from 37–97P **(red)** and 37–97S **(blue)**.

Additionally, DNA samples from penicillin-persister (37–97P) and susceptible cells of the strain 37–97 (37–97S) underwent whole-genome sequencing (WGS). Both genomes have a GC content of 38.5% and 1.92 Mb in size. More details on the genome attributes for 37–97S (Acc: CP041408.1) and 37–97P (Acc: CP041615.1) are listed in the Supplementary Table 4. Both sequences were classified as ST62 by the MLST software. To calculate the ANI value, we used the genome sequence of a ST62 *S. pyogenes*, strain NGAS743, available in the GenBank (Acc: CP007560.1). The ANI value was 99.95% (coverage 37–97S = 78.66% and coverage NGAS743 = 78.83%). This value was higher than the optimal genome-wide ANI threshold for species delineation (ANI 95%; coverage 70%). WGS alignments generated in MAUVE showed high identity and perfect synteny of collinear blocks (Figure 2A). There was also no difference in the absence or presence of mobile genetic elements, genomic islands, or unique genes in the persister cells of the strain 37–97 (37–97P) compared with that of susceptible ones (37–97S). The ANI value for the genomes of 37–97S and 37–97P was 99.99% (coverage 37–97S = 99.89% and coverage 37–97P = 99.89%). Despite some differences in SNPs observed in the WGS, these could not be confirmed by Sanger resequencing of these regions, thus mutations were not associated with the emergence of persisters (Figure 2B) ruling out the phenomenon known as SCVs.

### Proteomic Analysis

**FIGURE 3:**
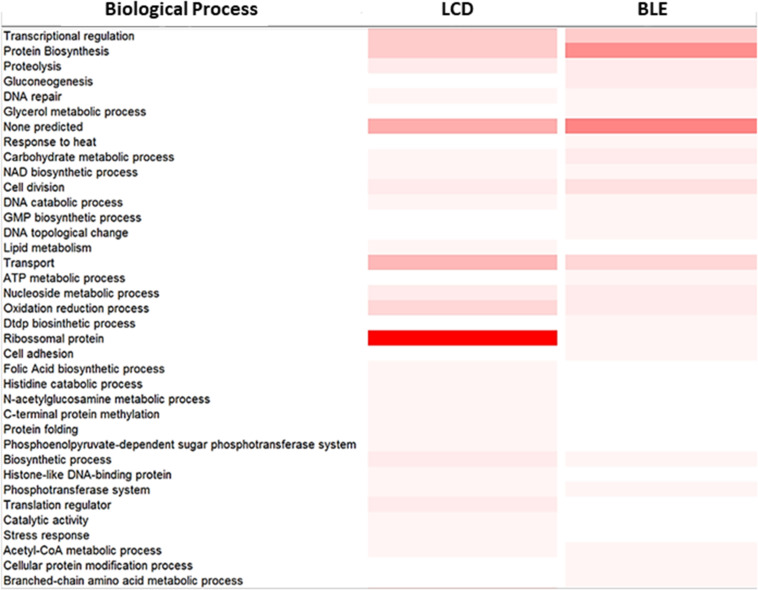
Proteomic analysis of the *Streptococcus pyogenes* strain 37–97 in biofilm-like environment (**BLE**) and at condition of low cell density (**LCD**). Color gradient indicates the frequency of proteins involved in a biological process according to InterPro (www.ebi.ac.uk/interpro). *More intense color*: Highest number of proteins. *Lightest color*: smallest number of proteins. *White color*: absence of proteins.

A total of 61 proteins were only detected in bacterial cells recovered from the biofilm-like environment of the strain 37–97, a condition that led to the formation of antimicrobial persisters (Supplementary Table 5). The most remarkable feature was the low frequency of L ribosomal proteins (LRP) in these GAS cells (3.3%). Seventy-nine proteins were only detected when GAS strain 37–97 was grown using an inoculum size recommended for MIC determination (Supplementary Table 6), a condition for susceptibility. However, the most frequently detected proteins under this condition were the LRP proteins (31.6%), which play essential roles in ribosome assembly and are crucial for protein synthesis and cell growth (Figure 3). These data clearly suggest a decrease in growth activity for cells grown in biofilm-like environments. Some multidrug resistance (MDR) efflux pump components were only detected under condition of cell agglomeration, including a protein associated with the periplasmic component of the efflux system that belongs to the root-nodulation-cell-division (RND) family (Uniprot access: Q1J790). Multiple sugar transport ATP-binding protein MsmK (Uniprot access: Q1J4L0) and the multidrug resistant ABC transporter ATP-binding and permease protein (Uniprot access: Q1J8L9) were also observed under this condition (Supplementary Table 5). A total of 128 proteins were detected in both conditions (Supplementary Table 7).

### Implication of Efflux Pumps

**FIGURE 4:**
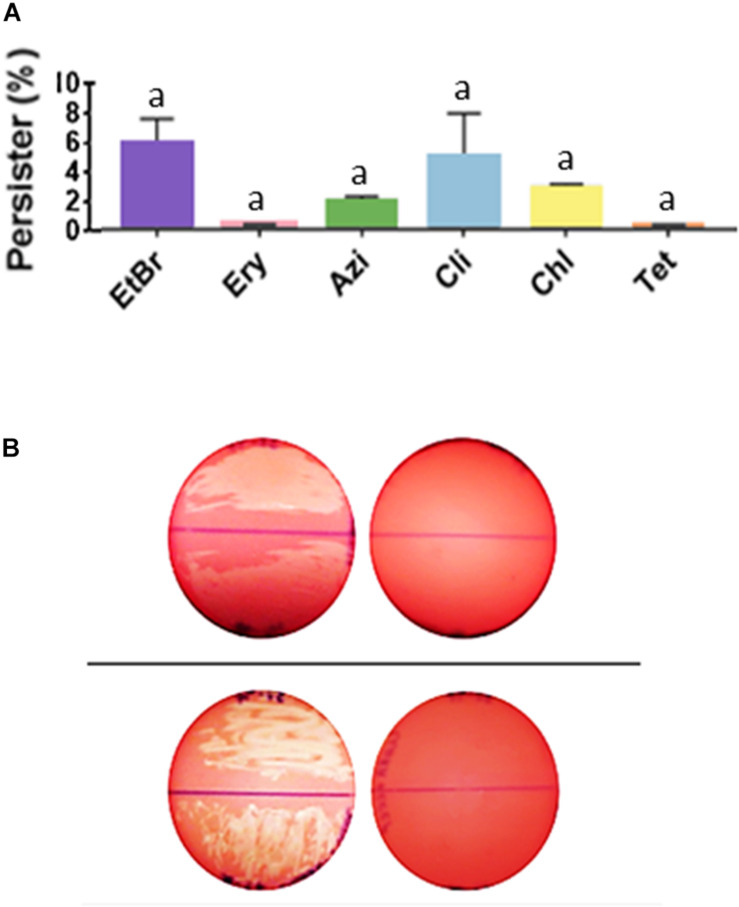
*Streptococcus pyogenes* cells in biofilm-like environments show persistence to ethidium bromide and to a number of non-β-lactam antimicrobials. **(A)** Persisters recovered from biofilm-like environments at concentrations of 4 μg/mL ethidium bromide (EtBr; MIC = 0.06 μg/mL), 4 μg/mL erythromycin (Ery; MIC = 0.12 μg/mL), 4 μg/mL azithromycin (Azi; MIC = 0.12 μg/mL), 1 μg/mL clindamycin (Cli; MIC = 0.01 μg/mL), 16 μg/mL chloramphenicol (Chl; MIC = 1 μg/mL), or 16 μg/mL tetracycline (Tet; MIC = 0.12 μg/mL). The average CFU/mL of the control cells (no antibiotic) was 5.5 × 10^10^ and corresponded to 100%. One way ANOVA was applied using CFU values (*p* < 0.001; *F* = 24.55; DF_to__tal_ = 37). *Post hoc* Tukey’s test followed ANOVA was performed for multiple comparisons between the control and each condition (^a^*p* < 0.001) and between the different conditions (there was no significant difference in the amount of persister cells recovered, *p* range = 0.995 to > 0.999). **(B)** Inhibition of antimicrobial persisters by the efflux pump inhibitor, cyanide 3-chlorophenylhydrazone (CCCP). *Top panel*; *left plate*: uncovered BAB plate was supplemented with 1 μg/mL Cli, and *right plate*: with 1 μg/mL Cli and 100 μM/mL CCCP. *Bottom panel*; *left plate*: uncovered BAB plate was supplemented with 16 μg/mL Chl, and *right plate*: with 16 μg/mL Chl and 100 μM/mL CCCP. Note the hemolysis that was produced by tiny (almost invisible) colonies for GAS cells in biofilm-like environments. Persisters for these antibiotics was completely inhibited in the presence of CCCP.

Efflux pump substrates (EtBr and different classes of antimicrobials) were used to assess the role of efflux pump activity in the formation of persisters. The MIC value of strain 37–97 for EtBr was 0.06 μg/mL. However, in the condition used to allow persister formation, growth was observed at concentrations of EtBr as high as 4 μg/mL, possibly indicating intense efflux activity. The percentage of EtBr-refractory cells recovered at concentration of 4 μg/mL was about 6% (*p* = 0.001) of the GAS cell population grown in biofilm-like environments in the absence of EtBr (5.5 ± 2.0 × 10^10^ CFU/mL) (Figure 4A).

Our data show that persisters were formed not only for the GAS representative strain 37–97 but similarly for the nine additional strains used as control, independent on the antibiotic classes analyzed, demonstrating that this phenomenon is a common feature in *S. pyogenes.* Persister cells were generated at MIC levels and at concentrations as high as 4 μg/mL erythromycin (MIC = 0.12 μg/mL), 4 μg/mL azithromycin (MIC = 0.12 μg/mL), 1 μg/mL clindamycin (MIC = 0.01 μg/mL), 16 μg/mL chloramphenicol (MIC = 1 μg/mL), and 16 μg/mL tetracycline (MIC = 0.12 μg/mL). The percentage of persisters recovered for 37–97 strain, considering all antimicrobials tested, ranged from 0.32 to 4.62% (*p* < 0.001) of the cell population grown in the absence of antimicrobials (5.5 ± 2.0 × 10^10^ CFU/mL) (Figure 4A).

Since the resistance-nodulation-division (RND) family of efflux pumps was one of the drug/proton antiporters detected in the proteome performed with cell grown under agglomeration condition, we used the pump inhibitor CCCP to dissipate the proton-motive force. Control plates with CCCP (100 μM) without antibiotic caused no effect on bacterial growth of 37–97. Despite the inhibition of chloramphenicol and clindamycin persisters by CCCP (Figure 4B), this compound did not inhibit the generation of persisters by β-lactams or other antimicrobials tested.

### Gene Expression Analysis

**FIGURE 5:**
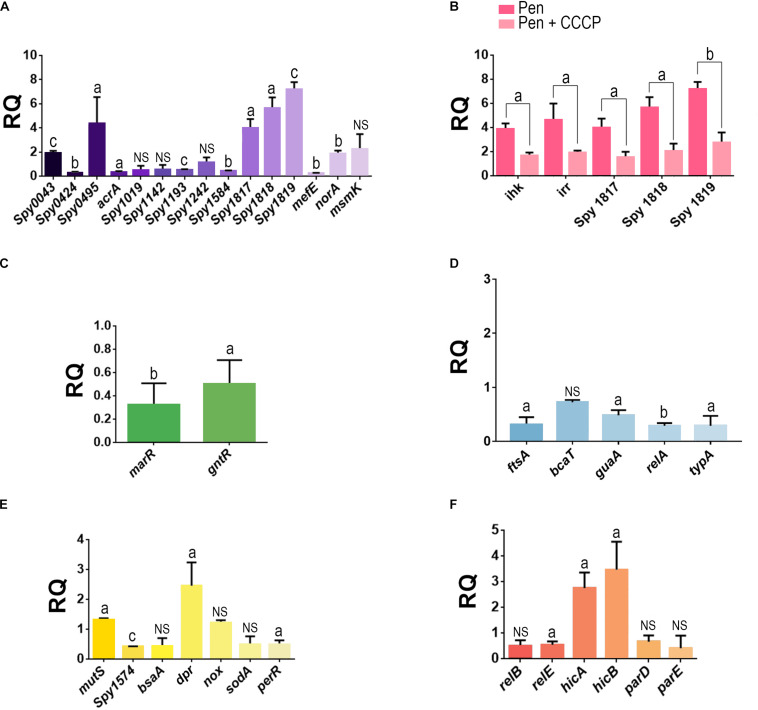
Analysis of the gene expression in penicillin-persister cells of the *Streptococcus pyogenes* strain 37–97. **(A)** Transcript levels of genes associated with efflux pumps in penicillin persisters. **(B)** Influence of CCCP in the expression of genes associated with ABC-type efflux pump (Locus_tag: *MGAS10750_Spy1817*, *MGAS10750_Spy1818*, and *MGAS10750_Spy1819*) and *ihk/irr* regulators in penicillin persisters. **(C)** Transcript levels of genes associated with negative transcriptional regulators of efflux pump in penicillin persisters. **(D)** Transcript levels of genes associated with protein biosynthesis and cell growth in penicillin-persisters. **(E)** Transcript levels of genes associated with stress conditions in penicillin-persisters. **(F)** Transcripts levels of genes associated with toxin-antitoxin (TA) systems in penicillin-persisters. Gene locus tags annotated in the genome of the strain MGAS10750 (Acc: NC_008024) were used as a reference sequences for primer design. Student’s *t*-test was applied. ^*a*^*p* < 0.05, ^*b*^*p* < 0.01, ^*c*^*p* < 0.001. For all gene expression assays, the respective calibrator sample (susceptible cells) was assigned relative quantification equal 1. For all tests, JZS Bayes factor agrees with Student’s *t*-test, except for the gene locus *MGAS10750_*S*py1019* for which the Bayes Factor (BF = 1.042144) was in favor of the alternative hypothesis, *bsaA* (BF = 1.289759, in favor of alternative hypothesis), *sodA* (BF = 1.213086, in favor of the alternative hypothesis), and *relB* (BF = 1.349216, in favor of the alternative hypothesis). RQ, Relative quantification.

Of the 15 genes analyzed that were associated with the efflux pumps, seven showed some levels of upregulation in penicillin-persister cells compared with those of susceptible GAS cells (Figure 5A). Among these, genes of an operon associated with efflux pumps of the RND family showed increases of ≥4-fold, which included *MGAS10750_Spy1817* (gene product: ABC transporter ATP binding protein; *p* = 0.0156), *MGAS10750_Spy1818* (gene product: ABC transporter permease protein; *p* = 0.0088), and *MGAS10750_Spy1819* (gene product: periplasmic component of efflux system, *p* < 0.001). An increase in transcripts >4-fold was also observed for a gene product annotated as belonging to a major facilitator superfamily, the multidrug resistance protein B (*MGAS10750_Spy0495*) (*p* = 0.04). Another gene upregulated was a homolog of the multiple sugar transport ATP-binding protein MsmK (*MGAS10750_Spy1776*), which displayed a 2.2-fold increase in expression levels. Despite this difference was not very expressive (*p* = 0.1490), protein of this same family was detected only on the proteome of cells grown in the biofilm-like environment. The loci *MGAS10750_Spy0043* and *MGAS10750_Spy1633* (*norA* homolog) showed about twofold increase (*p* < 0.001 and *p* = 0.0031, respectively, Figure 5A).

Because clindamycin was one of the antibiotics completely inhibited by CCCP, we also investigated the effect of this antibiotic in the overexpression of *MGAS10750_Spy1819*, which is part of the ABC transport operon. Our data showed an overexpression of about ninefold in these gene transcripts. It was observed that CCCP had simultaneously affected the transcript levels of this ABC operon and two gene homologs to *ihk* (*MGAS10750_Spy1815*) and *irr* (*MGAS10750_ Spy1816*) encoding the two-component regulatory system (TCS) Ihk/Irr, which are adjacent to and upstream this operon. Our data also demonstrated that both the operon and TCS were downregulated in the presence of CCCP (ABC operon: *p* = 0.03, *p* = 0.021, *p* < 0.001 and *ihk/irr*: *p* = 0.0057, *p* = 0.0389, respectively, Figure 5B). Similar to the downstream genes belonging to the ABC operon, *ihk/irr* homologs also displayed increased levels of transcripts (fourfold) for persisters formed in the absence of CCCP (*p* < 0.001 and *p* = 0.01), compared with the susceptible GAS cells (Figure 5B). These data suggest that the *Ihk/Irr* system could be acting as a regulator of this operon. Indeed, consistent with an increase in pump activity, genes (M*GAS10750_Spy1765* and M*GAS10750_Spy1120*) annotated as belonging to the MarR and GntR families (pump negative transcriptional regulators) were downregulated in the persister cells (*p* = 0.0032 and *p* = 0.0137; Figure 5C).

Additionally, the expression of genes associated with protein biosynthesis and cell growth/division were evaluated. For all these genes, the transcript levels decreased, but for *bcaT* homolog (gene product: branched-chain-amino acid aminotransferase) this decrease was less than twofold. The *guaA* homolog (gene product: GMP synthesis [glutamine hydrolyzing]), which is involved in the GTP pathway, was twofold down-regulated (*p* = 0.016). Decreased expression was also observed for *relA* (gene product: GTP pyrophosphokinase; *p* = 0.003) and *typA* (gene product: GTP-binding protein TypA/BipA; *p* = 0.0314). Finally, the *ftsA* homolog, which is essential for cell division, was reduced threefold (*p* = 0.0187) (Figure 5D).

Among the genes associated with the stress conditions studied, which includes some genes related to oxidative stress, the majority was down-regulated in penicillin-persisters. An increase was only observed for a *dpr* homolog (gene product: hydrogen peroxide resistance regulator), which was about twofold (*p* = 0.03) more expressed compared with the susceptible GAS cells (Figure 5E). Finally, we examined the expression of three genes homologous to toxin-antitoxin (TA) systems found in the genome of *S. pyogenes* strain 37–97. Increased expression was observed for the *hicA/B* homologs (2.8- and 3.5-fold increase, respectively; *p* = 0.007 and *p* = 0.017, respectively) for persisters (Figure 5F).

## Discussion

The influence of high bacterial load in environments such those found in biofilms on antimicrobial persistence *in vitro* and *in vivo* has been described by others (Thulin et al., 2006; Rio-Marques et al., 2014; Karslake et al., 2016; Li et al., 2017; Vulin et al., 2018; Novais et al., 2020). Persisters have conventionally been detected by indirectly determining CFUs after treating the bacterial cells with a high concentration of an antibiotic, or from bacterial cells that do not grow in the presence of the antibiotic, but regrow under a microfluidic device after drug removal (Orman and Brynildsen, 2015; Yu et al., 2019). Recently, Yu et al. (2019), detected persisters not only from 24 h stationary phase cultures treated with antibiotics but also observed increased detection of persisters from 12 to 24 h incubation, demonstrating the heterogeneous nature of the phenomenon. Additionally, their studies suggested that multiple proteins, important for cell growth, are sequestered in reversible subcellular structures, named regrowth-delay bodies, in non-growing cells. Notably, they also demonstrated that different depths of persistence occur in persister cells (Yu et al., 2019). In our study, *S. pyogenes* persisters were developed during antibiotic exposition of a high bacterial load placed onto the surface of cellophane membranes covering BAB plates. Although the last phase of biofilm accumulation does not occur in this model, due to the presence of antibiotics, the cell accumulation in the initially formed bacterial film (due the high bacterial load applied onto the membrane surface) led to generation of GAS persisters by possibly inducing growth impairment, as demonstrated by the proteome and gene expression data.

Despite the fact that it might be a reason for failures in the drug therapy, antimicrobial persistence remains unexplored in *S. pyogenes*. Here, we showed that GAS cells in an agglomerated condition persist not only to β-lactams but also to various classes of antimicrobials, corresponding to about 0.3–6.0% of the total bacterial population, depending on the drug tested. It is important to emphasize that drug persistence was not a particular characteristic of only one or few representatives of GAS since different isolates with distinct PFGE patterns were tested.

Phenotypic reversion was observed, indicating the involvement of non-inherited antimicrobial resistance mechanisms. Also, no mutation was detected in the tiny colonies formed by persister cells. In addition, the colony size returned to normal after growth in antibiotic-free medium, discarding the phenomenon of SVCs (Proctor, 2019; Huemer et al., 2020). In addition, no heterogeneous subpopulation displaying distinct penicillin MIC value was detected in the cell culture of the 37–97 strain ruling out heteroresistance phenotype. Also, penicillin tolerance was not observed for this strain (MCB/MIC = 1) (Melo et al., 2003). It is important to note that Vulin et al. (2018) found that various environmental signals might trigger the entry of *S. aureus* into a phenotypic state of growth arrest, including high bacterial density. Corroborating our finds, they found that persisters formed tiny colonies similar to SCVs that reverted to normal size after regrowth in fresh media (instable SVC phenotype). Also, they clearly demonstrated, using live-imaging microscopy, that persisters showed lag-phase delay and that antibiotics can even increase the proportion of instable SCV phenotypes.

There is no question that bacterial resistance acquired through genetic mechanisms is the major reason for clinical failures during antimicrobial therapy for many other pathogens. However, the importance of non-inherited resistance should not be disregarded, mainly concerning infections affecting immunocompromised patients, those associated with biofilm production, or severe and invasive infections where high number of bacterial cells can accumulate at the site of infection (Thulin et al., 2006).

It is remarkable that high bacterial load was detected in tissue biopsy specimens from 17 patients presenting with GAS disseminated infections (necrotizing fasciitis or severe cellulitis) despite intravenous antibiotic therapy (clindamycin in combination with β-lactam antibiotic) for a prolonged time (Thulin et al., 2006). Those authors suggested that GAS survival inside macrophages could represent a mechanism preventing bacterial eradication. However, patterns of purely intracellular bacteria were observed in less than half of the biopsies analyzed (Thulin et al., 2006). Some *in vitro* studies have demonstrated the effect of biofilm and high cell density in the failure of antibiotics to eliminate *Escherichia coli*, mycobacteria and methicillin-resistant *S. aureus* (Nielsen et al., 2011; Ferro et al., 2015; Coates et al., 2018; Novais et al., 2020). In fact, our findings demonstrated the generation of antimicrobial persisters for GAS in an agglomerated cell environment, which was associated with inhibition of both protein biosynthesis and cell growth, and possibly with an increased activity of intrinsic multidrug-resistant (MDR) efflux pumps.

It was observed that CCCP fully restored the susceptibility to clindamycin and chloramphenicol, suggesting the involvement of proton efflux pumps in GAS persistence/refractory to these drugs. In fact, a gene of the ABC operon of the RND family (that uses proton gradient force across inner membrane to exclude drugs; Eicher et al., 2014) was almost ninefold overexpressed in GAS-persister cells induced by clindamycin, and was detected only in the proteome of cells grown in biofilm-like environments. It is possible that additional efflux pumps, not importantly affected by CCCP, may be involved in the extrusion of the other antimicrobials tested. This assumption is supported by the fact that CCCP did not recover GAS susceptibility to the pump substrate EtBr. Indeed, about 50% of the efflux-associated genes analyzed were upregulated in penicillin-persister cells.

Typically, overexpression of efflux pumps confers resistance to different classes of antimicrobial agents and some dyes, such as EtBr, in other bacterial species (DeMarco et al., 2007; Martins et al., 2011; Sun et al., 2014; Wang et al., 2019). The involvement of conserved RND proteins in reducing *S. aureus* persistence to β-lactams and glycopeptides has also been demonstrated (Quiblier et al., 2011). Similar to our findings, Poudyal, and Sauer found increased expression of genes associated with an ABC transporter and other transport systems in *Pseudomonas aeruginosa* grown in biofilm conditions, suggesting that these mechanisms contributed to the persister phenotype of *P. aeruginosa* to tobramycin (Poudyal and Sauer, 2017). In fact, in our study, a homolog of *marR*, a negative pump regulator, was down-regulated in the persisters. In line with these data, increased resistance in *Burkholderia thailandensis* was attributed to enhanced efflux pump activity and was detected after repression of a *marR* homolog (Sabrin et al., 2019). Additionally, we found that a gene in the GntR family of regulators was also down-regulated in penicillin-persister GAS cells. It is remarkable that a *norG* knockout in *S. aureus* (a member of the GntR family) led to a threefold increase in the expression of an *abcA* gene encoding a protein of the ABC transport system with a concomitant increase in resistance to β-lactams (Truong-Bolduc and Hooper, 2007).

The Ihk/Irr two-component system is involved in the regulation of various streptococcal processes, including virulence (Han et al., 2012; Kachroo et al., 2020). It is notable that *ihk*/*irr* were overexpressed in a non-human primate model of GAS necrotizing myositis, and these genes were implicated in GAS resistance to polymorphonuclear phagocytosis (Kachroo et al., 2020). The fact that CCCP inhibited *ihk*/*irr* gene regulators and the RND family operon concomitantly, and that both the operon and *cis* regulators displayed increased expression in GAS-persister cells, raises an interesting hypothesis about another possible role for the Ihk/Irr system beyond virulence regulation. However, despite the genes co-localization and the concomitant downregulation by CCCP of *ihk*/*irr* and genes of the ABC transport operon, molecular cloning strategies are needed to validate the hypothesis that *ihk/irr* may not only regulate GAS virulence but also this transport system in 37–97 strain. Corroborating this hypothesis, microarrays data from Voyich et al. (2004) obtained from an *irr* mutant of the GAS strain, JRS500, revealed that a number of ABC transport genes were downregulated in this mutant, as well as the *msmK* gene.

Oxidative stress has also been associated with antimicrobial persisters in *E*. *coli* (Wu et al., 2012). In this study, a *dpr* homolog was upregulated in penicillin-persister GAS cells. The *dpr* gene encodes the non-specific DNA binding protein Dps (peroxide resistance protein, Dpr), and homologs have been identified in different bacterial species associated with protection against multiple stressors (Leszczynska et al., 2013). Dps protein forms self-aggregates and an insoluble complex with DNA. In *E. coli*, the aggregates formed in the stationary growth phase correlated with increased persisters formation (Leszczynska et al., 2013). Also, the induction of *dps* in *E. coli* resulted in overexpression of the toxin/antitoxin (TA) system MqsR/MqaA (Kim et al., 2010). In fact, in our study, the TA system of the HicAB family was upregulated in GAS-persister cells. However, the contribution of TA system and (p)ppGpp in *E. coli* persisters remains controversial (Goormaghtigh et al., 2018).

In addition to efflux pumps, stress conditions, and TA systems, slow-growing cells and stringent responses have also been implicated in the generation of antimicrobial persisters (Goormaghtigh et al., 2018; Vulin et al., 2018). The GAS persisters produced tiny colonies, indicating a condition of slow growth. Actually, genes associated with protein biosynthesis were downregulated in the penicillin-persisters, including homologs of *typA*/*bipA* (important in ribosome assembly) and *guaA* (essential in GTP synthesis). The inhibition of *guaBA* operon by (p)ppGpp in *E. coli* led to low levels GTP and increased bacterial survival during amino acid starvation (Hauryliuk et al., 2015). These results agree with the proteomic data that showed increased expression of ribosome protein L in GAS cells grown at low density population (susceptible cells) compared with cells obtained from the biofilm-like environment. Indeed, the expression of the *ftsA* gene, which is essential for cell growth, was reduced in penicillin-persister GAS cells. It is notable that a substantial reduction in transcription and translation of this gene was previously observed for antimicrobial-persisters in *E. coli*, which was associated with increased expression of different RNases (including RNase E, which is involved in the specific degradation of *ftsA*-*ftsz* transcripts) (Radzikowski et al., 2016). Indeed, *ftsA* and *ftsZ* proteins are among the proteins sequestered in regrowth-delay bodies found in *Shigella flexineri* and *Salmonella* Thyphimurium persisters forming non-growing cells (Yu et al., 2019).

Bacterial persistence to antibiotics is still a controversial issue that has been attributed to several mechanisms. The discrimination between the different phenomena does not seem to be an easy task. However, the antimicrobial persistence observed in our study could not be classified as heteroresistance, tolerance or stable SCVs phenotypes and are better defined as persisters on the basis of the following features: i. the level of persistence is not greatly affected by antibiotic concentrations since persisters can grow in antibiotic levels far above the MIC; ii. when regrown in the absence of the antimicrobial, persisters completely restore drug susceptibility to MIC values; iii. not all bacterial cells in the culture are killed at the same frequency; iv. they frequently exhibit persistence to different classes of antibiotics; v. the advantage of persisters against the susceptible cells, in the bacterial population, seems to be the slow-growing/non-growing characteristic of the persister cells (Vulin et al., 2018; Yu et al., 2019; Pontes and Groisman, 2019).

In conclusion, we showed that subpopulations of GAS cells can become persistent to high concentrations of β-lactams and other antimicrobials when cells in condition of agglomeration such as those observed in high bacterial load and biofilm environments formed on biotic or abiotic surfaces. Our data suggest that growth arrest and efflux pump are mechanisms associated with this phenotypic resistance in GAS cells, which have also been observed for persisters formed by other bacterial species (Poudyal and Sauer, 2017; Vulin et al., 2018; Pontes and Groisman, 2019). It is possible that this phenomenon might have some implications for failures in antimicrobial therapy that have been reported for some GAS clinical infections (Gidengil et al., 2013; Brook, 2017), including those severe and sometimes lethal invasive diseases, for which high bacterial load (∼10^7^ CFU/cm^2^) can be achieved in the infected tissues, despite the use of effective antimicrobial therapy (Thulin et al., 2006), and should not be overlooked. Finally, further studies with *ihk*/*irr* are required to clarify the role of this TC system in antimicrobial clinical failures due to a possible upregulation of both intracellular survival of GAS into macrophages and efflux pump activity.

## Data Availability Statement

The datasets presented in this study can be found in online repositories. The names of the repository/repositories and accession number(s) can be found below: https://www.ncbi.nlm.nih.gov/genbank/, CP041615.1 and https://www.ncbi.nlm.nih.gov/genbank/, CP041408.1.

## Ethics Statement

The studies involving human participants were reviewed and approved by the Ethics Committee of the Hospital Universitario Clementino Fraga Filho UFRJ/RJ under the # 4-485-002; the study was considered nonhuman subject research.

## Author Contributions

AF, BF-C, and RZ performed the conception and design of the work. AC, CM, CG, MMa, MMe, TA, and ÚL carried out the experiments. AF, AC, BF-C, CM, and RZ analyzed and interpreted the data. AB, AV, LA, and PP performed the whole genome sequencing and analysis. AF, BF-C, and CM wrote and revised the manuscript. All authors read and approved the final manuscript.

## Conflict of Interest

The authors declare that the research was conducted in the absence of any commercial or financial relationships that could be construed as a potential conflict of interest.

## Publisher’s Note

All claims expressed in this article are solely those of the authors and do not necessarily represent those of their affiliated organizations, or those of the publisher, the editors and the reviewers. Any product that may be evaluated in this article, or claim that may be made by its manufacturer, is not guaranteed or endorsed by the publisher.

## Footnotes

1
http://blast.ncbi.nlm.nih.gov/Blast.cgi

2
www.uniprot.org/blast

3
www.phrap.org/phrap.docs/phrap.html

4
https://www.ezbiocloud.net/tools/ani

5
https://cge.cbs.dtu.dk/services/MLST/

## Supplementary Material

Supplementary Table 1 Isolation year, geographic region, clinical origin of the 211 *Streptococcus pyogenes* isolates used in this study.

Supplementary Table 2 Characteristics of the *Streptococcus pyogenes* strains selected as controls for the experiments of persister generation for different classes of antimicrobials.

Supplementary Table 3 Primers used for real-time quantitative reverse transcriptase PCR (real-time RT-qPCR).

Supplementary Table 4 Characteristics of the *Streptococcus pyogenes* genomes sequenced.

Supplementary Table 5 Proteins identified in *Streptococcus pyogenes* strain 37–97 grown in biofilm-like environments.

Supplementary Table 6 Proteins identified in *Streptococcus pyogenes* strain 37–97 grown using low cell density.

Supplementary Table 7 Proteins identified in *Streptococcus pyogenes* strain 37–97 grown in both biofilm-like environments and using low cell density.
