# Supplementary material for: Cellular Growth Arrest and Efflux Pumps Are Associated With Antibiotic Persisters in Streptococcus pyogenes Induced in Biofilm-Like Environments
Source: Front Microbiol. 2021 Sep 21;12:716628. doi: 10.3389/fmicb.2021.716628 (PMC8490960; doi:10.3389/fmicb.2021.716628)
Supplement: Supplementary Table 1 — Isolation year, geographic region, clinical origin of the 211 Streptococcus pyogenes isolates used in this study. [file Data_Sheet_1.PDF]

Supplementary Table S1. Isolation year, geographic region, clinical origin of the 211 *Streptococcus pyogenes* isolates used in this study.

Strain	Year	City/State[a]	Infection/Clinical source	Patient type
1-78	1978	Rio de Janeiro, RJ	impetigo	outpatient
2-78	1978	Rio de Janeiro, RJ	impetigo	outpatient
4-78*	1978	Rio de Janeiro, RJ	oropharynx[b]	outpatient
5-78*	1978	Rio de Janeiro, RJ	oropharynx	outpatient
6-78	1978	Rio de Janeiro, RJ	impetigo	outpatient
7-78	1978	Rio de Janeiro, RJ	impetigo	outpatient
8-78*	1978	Rio de Janeiro, RJ	oropharynx	outpatient
9-78*	1978	Rio de Janeiro, RJ	oropharynx	outpatient
10-78*	1978	Rio de Janeiro, RJ	oropharynx	outpatient
11-78	1978	Rio de Janeiro, RJ	impetigo	outpatient
1-79	1979	Rio de Janeiro, RJ	impetigo	outpatient
2-79*	1979	Rio de Janeiro, RJ	oropharynx	outpatient
4-79	1979	Rio de Janeiro, RJ	impetigo	outpatient
5-79	1979	Rio de Janeiro, RJ	impetigo	outpatient
6-79	1979	Rio de Janeiro, RJ	impetigo	outpatient
7-79	1979	Rio de Janeiro, RJ	impetigo	outpatient
8-79*	1979	Rio de Janeiro, RJ	oropharynx	outpatient
9-79*	1979	Rio de Janeiro, RJ	oropharynx	outpatient
10-79*	1979	Rio de Janeiro, RJ	oropharynx	outpatient
11-79	1979	Rio de Janeiro, RJ	impetigo	outpatient
12-79	1979	Rio de Janeiro, RJ	abscess secretion	inpatient
1-80	1980	Rio de Janeiro, RJ	impetigo	outpatient
2-80	1980	Rio de Janeiro, RJ	impetigo	outpatient
3-80	1980	Rio de Janeiro, RJ	impetigo	outpatient
4-80	1980	Rio de Janeiro, RJ	impetigo	outpatient
5-80	1980	Rio de Janeiro, RJ	impetigo	outpatient
6-80	1980	Rio de Janeiro, RJ	impetigo	outpatient
7-80*	1980	Rio de Janeiro, RJ	oropharynx	outpatient
8-80**	1980	Rio de Janeiro, RJ	oropharynx	outpatient
9-80*	1980	Rio de Janeiro, RJ	oropharynx	outpatient
10-80*	1980	Rio de Janeiro, RJ	oropharynx	outpatient
11-80**	1980	Rio de Janeiro, RJ	oropharynx	outpatient
12-80**	1980	Rio de Janeiro, RJ	oropharynx	outpatient
2-81*	1981	Rio de Janeiro, RJ	oropharynx	outpatient
3-81*	1981	Rio de Janeiro, RJ	oropharynx	outpatient
4-81*	1981	Rio de Janeiro, RJ	oropharynx	outpatient
5-81*	1981	Rio de Janeiro, RJ	oropharynx	outpatient
6-81*	1981	Rio de Janeiro, RJ	oropharynx	outpatient
7-81*	1981	Rio de Janeiro, RJ	oropharynx	outpatient
9-81*	1981	Rio de Janeiro, RJ	oropharynx	outpatient
10-81*	1981	Rio de Janeiro, RJ	oropharynx	outpatient
11-81*	1981	Rio de Janeiro, RJ	oropharynx	outpatient

Strain	Year	City/State[a]	Infection/Clinical source	Patient type
1-82*	1982	Araraquara, SP	oropharynx	outpatient
2-82*	1982	Araraquara, SP	oropharynx	outpatient
3-82*	1982	Araraquara, SP	oropharynx	outpatient
4-82*	1982	Araraquara, SP	oropharynx	outpatient
5-82*	1982	Araraquara, SP	oropharynx	outpatient
6-82*	1982	Araraquara, SP	oropharynx	outpatient
7-82*	1982	Araraquara, SP	oropharynx	outpatient
8-82*	1982	Araraquara, SP	oropharynx	outpatient
2-83*	1983	Rio de Janeiro, RJ	oropharynx	outpatient
3-83*	1983	Rio de Janeiro, RJ	oropharynx	outpatient
4-83*	1983	Rio de Janeiro, RJ	oropharynx	outpatient
1-84	1984	São Paulo, SP	impetigo	inpatient
2-84*	1984	Rio de Janeiro, RJ	oropharynx	outpatient
3-84	1984	São Paulo, SP	impetigo	inpatient
4-84**	1984	Rio de Janeiro, RJ	oropharynx	outpatient
5-84	1984	Rio de Janeiro, RJ	abscess secretion	inpatient
6-84	1984	São Paulo, SP	impetigo	inpatient
7-84	1984	São Paulo, SP	impetigo	outpatient
9-84	1984	São Paulo, SP	nasopharynx	outpatient
10-84*	1984	São Paulo, SP	oropharynx	outpatient
11-84*	1984	Rio de Janeiro, RJ	oropharynx	outpatient
1-85	1985	São Paulo, SP	impetigo	outpatient
2-85*	1985	Rio de Janeiro, RJ	oropharynx	outpatient
3-85	1985	São Paulo, RJ	impetigo	outpatient
4-85**	1985	Angra dos Reis, RJ	oropharynx	outpatient
5-85**	1985	Angra dos Reis, RJ	oropharynx	outpatient
6-85**	1985	Angra dos Reis, RJ	oropharynx	outpatient
7-85**	1985	Angra dos Reis, RJ	oropharynx	outpatient
8-85**	1985	Angra dos Reis, RJ	oropharynx	outpatient
9-85	1985	São Paulo, SP	impetigo	inpatient
10-85	1985	São Paulo, SP	impetigo	outpatient
11-85	1985	São Paulo, SP	impetigo	outpatient
12-85	1985	São Paulo, SP	impetigo	inpatient
1-86*	1986	Rio de Janeiro, RJ	oropharynx	outpatient
2-86**	1986	Rio de Janeiro, RJ	oropharynx	outpatient
3-86*	1986	Rio de Janeiro, RJ	oropharynx	outpatient
4-86*	1986	Rio de Janeiro, RJ	oropharynx	outpatient
5-86	1986	Ribeirão Preto, SP	impetigo	outpatient
6-86*	1986	Rio de Janeiro, RJ	oropharynx	outpatient
7-86*	1986	Rio de Janeiro, RJ	oropharynx	outpatient
8-86	1986	Ribeirão Preto, SP	impetigo	outpatient
9-86	1986	Ribeirão Preto, SP	pustule secretion	inpatient
10-86	1986	Ribeirão Preto, SP	abscess secretion	inpatient
1-87*	1987	Rio de Janeiro, RJ	oropharynx	outpatient
2-87*	1987	Rio de Janeiro, RJ	oropharynx	outpatient

Strain	Year	City/State[a]	Infection/Clinical source	Patient type
3-87*	1987	Rio de Janeiro, RJ	oropharynx	outpatient
4-87*	1987	Rio de Janeiro, RJ	oropharynx	outpatient
5-87*	1987	Rio de Janeiro, RJ	oropharynx	outpatient
6-87*	1987	Rio de Janeiro, RJ	oropharynx	outpatient
7-87**	1987	Rio de Janeiro, RJ	oropharynx	outpatient
11-87*	1987	Ribeirão Preto, SP	oropharynx	outpatient
12-87	1987	Ribeirão Preto, SP	impetigo	outpatient
14-87	1987	Ribeirão Preto, SP	abscess secretion	inpatient
15-87*	1987	Ribeirão Preto, SP	oropharynx	outpatient
16-87*	1987	Ribeirão Preto, SP	oropharynx	outpatient
17-87	1987	Rio de Janeiro, RJ	impetigo	outpatient
18-87	1987	Ribeirão Preto, SP	surgical wound	inpatient
19-87	1987	Ribeirão Preto, SP	pustule secretion	outpatient
20-87	1987	Ribeirão Preto, SP	sputum	inpatient
21-87	1987	Ribeirão Preto, SP	bacteremia	inpatient
24-87	1987	Ribeirão Preto, SP	impetigo	outpatient
1-88**	1988	Rio de Janeiro, RJ	oropharynx	outpatient
2-88**	1988	Rio de Janeiro, RJ	oropharynx	outpatient
3-88**	1988	Rio de Janeiro, RJ	oropharynx	outpatient
4-88**	1988	Rio de Janeiro, RJ	oropharynx	outpatient
6-88**	1988	Rio de Janeiro, RJ	oropharynx	outpatient
11-88	1988	Ribeirão Preto, SP	bacteremia	inpatient
12-88*	1988	Rio de Janeiro, RJ	oropharynx	outpatient
14-88	1988	Ribeirão Preto, SP	bacteremia	inpatient
15-88*	1988	Ribeirão Preto, SP	oropharynx	outpatient
16-88	1988	Ribeirão Preto, SP	perineal injury	inpatient
17-88	1988	Ribeirão Preto, SP	nasopharynx	outpatient
18-88*	1988	Ribeirão Preto, SP	oropharynx	outpatient
19-88	1988	Ribeirão Preto, SP	ascitic fluid	inpatient
21-88	1988	Ribeirão Preto, SP	breast secretion	inpatient
22-88	1988	Ribeirão Preto, SP	secretion	NR[c]
1-89**	1989	Rio de Janeiro, RJ	oropharynx	outpatient
2-89**	1989	Rio de Janeiro, RJ	oropharynx	outpatient
3-89**	1989	Rio de Janeiro, RJ	oropharynx	outpatient
4-89*	1989	Rio de Janeiro, RJ	oropharynx	outpatient
5-89**	1989	Rio de Janeiro, RJ	oropharynx	outpatient
7-89**	1989	Rio de Janeiro, RJ	oropharynx	outpatient
8-89**	1989	Rio de Janeiro, RJ	oropharynx	outpatient
16-89	1989	São Paulo, SP	NR	NR
17-89	1989	São Paulo, SP	NR	NR
18-89	1989	São Paulo, SP	NR	NR
19-89	1989	Ribeirão Preto, SP	abscess secretion	inpatient
20-89	1989	Ribeirão Preto, SP	ganglion secretion	inpatient
1-90**	1990	Rio de Janeiro, RJ	oropharynx	outpatient
2-90**	1990	Rio de Janeiro, RJ	oropharynx	outpatient

Strain	Year	City/State[a]	Infection/Clinical source	Patient type
3-90**	1990	Rio de Janeiro, RJ	oropharynx	outpatient
4-90	1990	Rio de Janeiro, RJ	impetigo	outpatient
5-90**	1990	Rio de Janeiro, RJ	oropharynx	outpatient
6-90**	1990	Rio de Janeiro, RJ	oropharynx	outpatient
7-90*	1990	Rio de Janeiro, RJ	oropharynx	outpatient
8-90*	1990	Rio de Janeiro, RJ	oropharynx	outpatient
9-90**	1990	Rio de Janeiro, RJ	oropharynx	outpatient
13-90	1990	Ribeirão Preto, SP	sputum	outpatient
14-90	1990	Ribeirão Preto, SP	abscess secretion	inpatient
17-90*	1990	Ribeirão Preto, SP	oropharynx	outpatient
18-90	1990	Ribeirão Preto, SP	bacteremia	inpatient
19-90	1990	Ribeirão Preto, SP	tracheal secretion	inpatient
4-91**	1991	Rio de Janeiro, RJ	oropharynx	outpatient
5-91**	1991	Rio de Janeiro, RJ	oropharynx	outpatient
6-91**	1991	Rio de Janeiro, RJ	oropharynx	outpatient
7-91**	1991	Rio de Janeiro, RJ	oropharynx	outpatient
8-91**	1991	Rio de Janeiro, RJ	oropharynx	outpatient
9-91**	1991	Rio de Janeiro, RJ	oropharynx	outpatient
10-91**	1991	Rio de Janeiro, RJ	oropharynx	outpatient
12-91	1991	Rio de Janeiro, RJ	impetigo	outpatient
13-91	1991	Ribeirão Preto, SP	bacteremia	inpatient
15-91	1991	São Paulo, SP	NR	NR
16-91	1991	São Paulo, SP	NR	NR
18-91	1991	São Paulo, SP	NR	NR
19-91	1991	São Paulo, SP	NR	NR
21-91	1991	Ribeirão Preto, SP	impetigo	outpatient
22-91	1991	São Paulo, SP	NR	NR
23-91	1991	São Paulo, SP	NR	NR
24-91	1991	Ribeirão Preto, SP	impetigo	outpatient
25-91	1991	São Paulo, SP	abscess secretion	inpatient
27-91	1991	Ribeirão Preto, SP	NR	NR
3-92**	1992	Rio de Janeiro, RJ	oropharynx	outpatient
4-92**	1992	Rio de Janeiro, RJ	oropharynx	outpatient
5-92**	1992	Rio de Janeiro, RJ	oropharynx	outpatient
6-92**	1992	Rio de Janeiro, RJ	oropharynx	outpatient
8-92*	1992	Rio de Janeiro, RJ	oropharynx	outpatient
9-92	1992	Florianópolis, SC	urethral secretion	inpatient
1-93	1993	Rio de Janeiro, RJ	impetigo	outpatient
2-93*	1993	Rio de Janeiro, RJ	oropharynx	outpatient
3-93*	1993	Rio de Janeiro, RJ	oropharynx	outpatient
4-93*	1993	Rio de Janeiro, RJ	oropharynx	outpatient
5-93*	1993	Rio de Janeiro, RJ	oropharynx	outpatient
6-93*	1993	Rio de Janeiro, RJ	oropharynx	outpatient
7-93*	1993	Rio de Janeiro, RJ	oropharynx	outpatient
9-93*	1993	Rio de Janeiro, RJ	oropharynx	outpatient

Strain	Year	City/State[a]	Infection/Clinical source	Patient type
10-96*	1996	Rio de Janeiro, RJ	oropharynx	outpatient
11-96*	1996	Rio de Janeiro, RJ	oropharynx	outpatient
17-96*	1996	Rio de Janeiro, RJ	oropharynx	inpatient
21-96*	1996	Duque de Caxias, RJ	oropharynx	outpatient
27-96	1996	Rio de Janeiro, RJ	tracheal secretion	inpatient
28-96	1996	Niterói, RJ	scarlet fever	inpatient
38-96*	1996	Rio de Janeiro, RJ	oropharynx	outpatient
41-96	1996	Rio de Janeiro, RJ	NR	NR
42-96	1996	Rio de Janeiro, RJ	wound secretion	outpatient
44-96	1996	Rio de Janeiro, RJ	impetigo	inpatient
45-96	1996	Niterói, RJ	erysipelas	outpatient
46-96	1996	Rio de Janeiro, RJ	vaginal secretion	outpatient
47-96	1996	Niterói, RJ	vaginal secretion	inpatient
49-96	1996	Rio de Janeiro, RJ	impetigo	outpatient
50-96	1996	Rio de Janeiro, RJ	abscess secretion	inpatient
51-96	1996	Rio de Janeiro, RJ	wound secretion	outpatient
52-96	1996	Niterói, RJ	pustule secretion	inpatient
1-97*	1997	Rio de Janeiro, RJ	oropharynx	outpatient
3-97	1997	Rio de Janeiro, RJ	NR	NR
7-97	1997	Rio de Janeiro, RJ	impetigo	outpatient
8-97*	1997	Rio de Janeiro, RJ	oropharynx	outpatient
11-97	1997	Niterói, RJ	abscess secretion	inpatient
13-97*	1997	Rio de Janeiro, RJ	oropharynx	outpatient
14-97*	1997	Rio de Janeiro, RJ	oropharynx	outpatient
15-97*	1997	Rio de Janeiro, RJ	oropharynx	outpatient
19-97	1997	Rio de Janeiro, RJ	wound secretion	inpatient
24-97	1997	Rio de Janeiro, RJ	NR	NR
26-97	1997	São João de Meriti, RJ	skin infection	outpatient
29-97*	1997	Niterói, RJ	oropharynx	outpatient
30-97	1997	Rio de Janeiro, RJ	urethral secretion	inpatient
34-97*	1997	Rio de Janeiro, RJ	oropharynx	outpatient
35-97*	1997	Rio de Janeiro, RJ	oropharynx	outpatient
36-97*	1997	Rio de Janeiro, RJ	oropharynx	outpatient
37-97*	1997	Rio de Janeiro, RJ	oropharynx	outpatient

[a] RJ, Rio de Janeiro; SP, São Paulo; SC, Santa Catarina

[b] The strain was obtained from the oropharynx of the patients with pharyngitis (*) or of carriers (**).

[c] NR, not reported.
