# Supplementary material for: Cellular Growth Arrest and Efflux Pumps Are Associated With Antibiotic Persisters in Streptococcus pyogenes Induced in Biofilm-Like Environments
Source: Front Microbiol. 2021 Sep 21;12:716628. doi: 10.3389/fmicb.2021.716628 (PMC8490960; doi:10.3389/fmicb.2021.716628)
Supplement: Supplementary Table 2 — Characteristics of the Streptococcus pyogenes strains selected as controls for the experiments of persister generation for different classes of antimicrobials. [file Data_Sheet_2.PDF]

Supplementary Table S2. Characteristics of the *Streptococcus pyogenes* strains selected as controls for the experiments of persister generation for different classes of antimicrobials.

**Strains**[a]	**Year**	**City/State**[b]	**Clinical source**	**PFGE pattern**[c]
2-78	1978	Rio de Janeiro/RJ	impetigo	C
44-96	1996	Rio de Janeiro/RJ	impetigo	AJ
21-87	1987	Ribeirão Preto/SP	bacteremia	AZ
18-90	1990	Ribeirão Preto/SP	bacteremia	AP
21-88	1988	Ribeirão Preto/SP	breast secretion	H
25-91	1991	São Paulo/SP	abscess secretion	AS
9-92	1992	Florianópolis/SC	urethral secretion	V
27-96	1996	Rio de Janeiro/RJ	tracheal secretion	AX
8-90	1990	Rio de Janeiro/RJ	oropharynx	M
37-97	1997	Rio de Janeiro/RJ	oropharynx	AA

[a]All strains were susceptible to penicillin; cephalexin; erythromycin; azithromycin; clindamycin; chloramphenicol and tetracycline (Melo et al., 2003).

[b]RJ, Rio de Janeiro; SP, São Paulo; SC, Santa Catarina.

[c] PFGE following genomic digestion of the isolates with the endonuclease *Sma*I was previously performed by Melo *et al*., 2003.

Note: Persisters were recovered from all *S. pyogenes* strains not only for β-lactams but also for other antimicrobials tested in concentrations far above the MICs. The strains 37-97 was randomly selected by sampling for the molecular studies.
