# Supplementary material for: Cellular Growth Arrest and Efflux Pumps Are Associated With Antibiotic Persisters in Streptococcus pyogenes Induced in Biofilm-Like Environments
Source: Front Microbiol. 2021 Sep 21;12:716628. doi: 10.3389/fmicb.2021.716628 (PMC8490960; doi:10.3389/fmicb.2021.716628)
Supplement: Supplementary Table 3 — Primers used for real-time quantitative reverse transcriptase PCR (real-time RT-qPCR). [file Data_Sheet_3.PDF]

Supplementary Table S3. Primers used for real-time quantitative reverse transcriptase PCR (real-time RT-qPCR).

Gene	Product	Class	Transporter family	Primer sequence (5' – 3')	Amplicon length (pb)	Reference
16S		Ribossomal		F: ACCAAGGCGACGATACATAG<br>R: GTGTCTCAGTCCCAGTGTG	61	This study
Spy0043	MATE family transporter	Transporter	Multidrug and toxic compound extrusion (MATE)	F: ACATTATCAGTATTTATCAGTCC<br>R: GACCGAAAGGAGGAGTGTTA	146	This study
Spy0424	Multi-drug resistance efflux pump	Efflux pump	Major Facilitator Superfamily (MFS)	F: CGCTTTGAGATGATACCTACA<br>R: TGGGGACTTTTTACCTGTTTTA	53	This study
Spy0495	Major facilitator superfamily protein	Transporter	Major Facilitator Superfamily (MFS)	F: ATGATGACCACTTCGGCTAC<br>R: AATGCCTTGAAAAATGCGGG	150	This study
acrA	ABC transporter substrate-binding protein	Transporter	ATP-binding cassette super family (ABC)	F: AGGCTTCACTGTTTCTGTTG<br>R: CATCTGCGTTTCCAAGTGTC	151	This study
Spy1019	Multidrug resistance ABC transporter ATP-binding and permease protein	Transporter	ATP-binding cassette super family (ABC)	F: TATGTGGTGCTATGGTAATG<br>R: GTAAGACAATCACCATCATC	78	This study
Spy1142	ABC transporter ATP-binding protein	Transporter	ATP-binding cassette super family (ABC)	F: ACCGCTTCAAAACTCTATCC<br>R: AATGTCCGAAATCAAGTGGG	203	This study
Spy1193	Major facilitator superfamily protein	Transporter	Major Facilitator Superfamily (MFS)	F: TCTTATTCGGGTTGCTGGCT<br>R: TATGGCAATGGCTACACCGT	179	This study
Spy1242	Major facilitator superfamily protein	Transporter	Major Facilitator Superfamily (MFS)	F: CAGGGTTTGCTATTATGGGG<br>R: CTATCTCAGCAGCAGTTGGTT	174	This study
Spy1584	ABC transporter ATP-binding protein	Transporter	ATP-binding cassette super family (ABC)	F: CTTTTTTAGAGAGATTTGCTGC<br>R: ACCTTATTGAACCGTAGCATT	61	This study
Spy1817	ABC transporter permease protein	Efflux pump	Permease	F: GCAAAGCGTGTCAGTGATAT<br>R: CATGACACCAAACATATAATCT	66	This study
Spy1818	ABC transporter ATP-binding protein	Efflux pump	ATP-binding cassette super family (ABC)	F: GAATATCATTGGGTGTTTAGAT<br>R: TAAGGATGACACGTCTCTGC	73	This study
Spy1819	Periplasmic component of efflux system	Efflux bomb	ATP-binding cassette super family (ABC)	F: GCGATGTCAAAACTGCTCAA<br>R: CCGTTGTGGCAGTAGCATTA	57	This study
mefE	Efflux protein	Efflux pump	Major Facilitator Superfamily (MFS)	F: GACGAAGTGGTGATAAAGGAG<br>R: GCCATAAAGTCCAGTTGAAAT	204	This study

Gene	Product	Class	Transporter family	Primer sequence (5’ – 3’)	Amplicon length (pb)	Reference
norA	Major facilitator transporter	Transporter	Major Facilitator Superfamily (MFS)	F: AGGCGATAAATGGGTCTTGTA<br>R: CTCCAGACGATACACTACCT	80	This study
msmK	Multiple sugar transport ATP-binding protein MsmK	Transporter	ATP-binding cassette super family (ABC)	F: CCAGTAACTCAGAAACCAA<br>R: AGTCACTTTTGGTATTCGTC	107	This study
gntR	GntR family transcriptional regulator	Transcriptional regulator	-	F: ATGTTCAATAGCGACGATAGTT<br>R: TCAGGGAAAAGCAATGGAAAA	194	This study
marR	Putative transcriptional regulator	Transcriptional regulator	-	F: ATGGTGGTGTTTCGTAAAGC<br>R: TATCAGCCCCAAAAGCATCTA	52	This study
ihk	Histidine kinase	Two-component		F: TCAGAACTAAAACACCTCAAAG<br>R: CTAGTTGGTTGTCTTTTAGTTG	163	This study
irr	Response regulatory	Two-component		F: GTTCGGTCTCATCAACAAGTA<br>R: GTGATTATGTGACCAAACCATT	123	This study
ftsA	Cell division protein	Cell division	-	F: GGTGTTAGTAATGTTCCAAGT<br>R: ACCGCAGTTTTGATGGCAGT	86	This study
bcaT	Branched-chain amino acid aminotransferase	Metabolism-related		F: CAACTATTTAGACCAGAC<br>R: ATTGCTCTGTTGATACTTGT	85	This study
guaA	GMP synthetase	Metabolism-related	-	F: GTGTTGACTTCCTTGCCCA<br>R: GCATTCCAAGAGCGATTCCA	190	This study
relA	GTP pyrophosphokinase activity	Metabolism-related		F: TACGGGGTTGCTGCTCACT<br>R: TTGACCTTACCACGCACGC	53	This study
typA	GTP-binding protein	Metabolism-related		F: CACTTCATCTACAACTTCTG<br>R: AGGAACAATGCCCCAGAC	124	This study
mutS	DNA mismatch repair	Stress-related	-	F: GCGGTTGTTGAAAAGGTTATG<br>R: CGCATCAAATAAAGGTAAATCA	204	This study
Spy1574	Universal stress protein	Stress-related	-	F: ACAAGCACAGATAGCAGGAAT<br>R: GAAGATGAGCCAATAAGGAGG	165	This study
bsaA	Glutathione peroxidase	Oxidative stress		F: AGTGAATGGTAAAGACGCTGA<br>R: AGGGGACCACTTTTTTCTTCT	63	This study
dpr	Non-specific DNA-binding protein Dps	Oxidative stress		F: GCTGTCGCTGACTTATCTGT<br>R:GCTATCAAGTAATTCATCCATTT	111	This study

Gene	Product	Class	Transporter family	Primer sequence (5' – 3')	Amplicon length (pb)	Reference
nox	NADH oxidase H2O-forming	Oxidative stress		F: TTATCCTCGCCGTTGGTTTC<br>R: CAAGGAAAGCACCATTACGG	93	This study
sodA	Superoxide dismutase	Oxidative stress		F: GGCAGATGTCACTAAGATTC<br>R: CAAAACGTCCAGTAGCAGC	125	This study
perR	Ferric uptake regulation protein	Oxidative stress		F: ATTTACCGTGACTTACAGCCT<br>R: CATAAAGTCATAGTAGGTGGT	140	This study
relB	Plasmid stabilization system antitoxin protein	TA system		F:ACTGCGATTGATATGTTTTTGAAT<br>R: CTGACATCCTGACGGTTGCC	175	This study
relE	Plasmid stabilization system toxin protein	TA system		F: GGCAGCGGATAATCTTCTTG<br>R: GTATAGCCTTTTCCAATCAGAT	107	This study
hicA	Toxin-antitoxin system, toxin component	TA system		F: CCCCAAAGCAGATGATAAAAC<br>R: TTTCCGAGGTCTTTATTGTGC	130	This study
hicB	Phage Protein/ Antitoxin	TA system		F: GCTACATTTACACAAGACACA<br>R: ACAGCCATTTCATAAGCCTCT	104	This study
parD	DNA-damage-inducible protein J/Antitoxin	TA system		F: GCGAAGCAATGACAGTATT<br>R: TCTTTCTCTAACTCCTCTTCC	127	This study
parE	Hypothetical cytosolic protein	TA system		F: CATTAGCGACATAGAAAAAC<br>R: TGAGTAGGAAGCAAGTAATC	186	This study
