# Supplementary material for: Cellular Growth Arrest and Efflux Pumps Are Associated With Antibiotic Persisters in Streptococcus pyogenes Induced in Biofilm-Like Environments
Source: Front Microbiol. 2021 Sep 21;12:716628. doi: 10.3389/fmicb.2021.716628 (PMC8490960; doi:10.3389/fmicb.2021.716628)
Supplement: Supplementary Table 4 — Characteristics of the Streptococcus pyogenes genomes sequenced. [file Data_Sheet_4.PDF]

Supplementary Table S4. Characteristics of the *Streptococcus pyogenes* genomes sequenced.

Name	Access number	Biosample	Size	G+C%	Protein	rRNA	tRNA	Other RNA	Gene	Genome coverage
37-97S[a]	CP041408.1	SAMN12251806	1.92	38.5	1,797	18	67	4	1,928	275.0x
37-97P[b]	CP041615.1	SAMN12251807	1.92	38.5	1,794	18	67	4	1,932	64.0x

[a]37-97S: DNA sample obtained from the susceptible cells of the *S. pyogenes* strain 37-97.

[b]37-97P: DNA sample obtained from penicillin-persister cells of the *S. pyogenes* strain 37-97.
