# Supplementary material for: Cellular Growth Arrest and Efflux Pumps Are Associated With Antibiotic Persisters in Streptococcus pyogenes Induced in Biofilm-Like Environments
Source: Front Microbiol. 2021 Sep 21;12:716628. doi: 10.3389/fmicb.2021.716628 (PMC8490960; doi:10.3389/fmicb.2021.716628)
Supplement: Supplementary Table 5 — Proteins identified in Streptococcus pyogenes strain 37–97 grown in biofilm-like environments. [file Data_Sheet_5.PDF]

Supplementary Table S5. Proteins identified in *Streptococcus pyogenes* strain 37-97 grown in biofilm-like environments.

Ac.No[a]	Protein name[b]
Q1J6B3_STRPF	NAD-dependent K+ or Na+ uptake system component / Transcriptional regulator, GntR family; *Streptococcus pyogenes* serotype M4 (strain MGAS10750)
Q1J8L3_STRPF	Glutamate tRNA ligase / gltx gene product - *Streptococcus pyogenes* serotype M4 (strain MGAS10750)
Q1J7X1_STRPF	Threonine--tRNA ligase**-** *Streptococcus pyogenes* serotype M4 (strain MGAS10750)
Q1J5E1_STRPF	Glycine -tRNA ligase beta subunit **-** *Streptococcus pyogenes* serotype M4 (strain MGAS10750)
Q1J433_STRPF	Endopeptidase degP (EC 3.4.21.-) - *Streptococcus pyogenes* serotype M4 (strain MGAS10750)
Q1J453_STRPF	L-serinedehydratase alpha subunit (EC 4.3.1.17) - *Streptococcus pyogenes* serotype M4 (strain MGAS10750)
Q1J454_STRPF	L-serinedehydratase beta subunit (EC 4.3.1.17) - *Streptococcus pyogenes* serotype M4 (strain MGAS10750)
Q1J489_STRPF	DNA mismatch repair protein mutS - *Streptococcus pyogenes* serotype M4 (strain MGAS10750)
Q1J4X6_STRPF	Dihydroxyacetone kinase family protein - *Streptococcus pyogenes* serotype M4 (strain MGAS10750)
Q1J528_STRPF	Xaa-Pro dipeptidase (EC 3.4.13.9) - *Streptococcus pyogenes* serotype M4 (strain MGAS10750)
Q1J579_STRPF	Chaperone protein dnaJ - *Streptococcus pyogenes* serotype M4 (strain MGAS10750)
Q1J591_STRPF	Biotin carboxylase (EC 6.3.4.14) - *Streptococcus pyogenes* serotype M4 (strain MGAS10750)
Q1J5D7_STRPF	Aldo/keto reductase family - *Streptococcus pyogenes* serotype M4 (strain MGAS10750)
Q1J5E4_STRPF	Glycerol kinase (EC 2.7.1.30) - *Streptococcus pyogenes* serotype M4 (strain MGAS10750)
Q1J5H0_STRPF	NH(3)-dependent NAD(+) synthetase (EC 6.3.5.1) - *Streptococcus pyogenes* serotype M4 (strain MGAS10750)
Q1J5H2_STRPF	Multimodular transpeptidase-transglycosylase PBP 1A - *Streptococcus pyogenes* serotype M4 (strain MGAS10750)
Q1J5I9_STRPF	Methionyl-tRNA formyltransferase (EC 2.1.2.9) - *Streptococcus pyogenes* serotype M4 (strain MGAS10750)
Q1J5S9_STRPF	Cell division protein FtsA - *Streptococcus pyogenes* serotype M4 (strain MGAS10750)
Q1J5U6_STRPF	Exodeoxyribonuclease 7 large subunit (EC 3.1.11.6) - *Streptococcus pyogenes* serotype M4 (strain MGAS10750)
Q1J5Z4_STRPF	Alanine-tRNA ligase (EC 6.1.1.7) - *Streptococcus pyogenes* serotype M4 (strain MGAS10750)
Q1J607_STRPF	Ribonucleoside-diphosphate reductase (EC 1.17.4.1) - *Streptococcus pyogenes* serotype M4 (strain MGAS10750)
Q1J631_STRPF	Methionine aminopeptidase (EC 3.4.11.18) - *Streptococcus pyogenes* serotype M4 (strain MGAS10750)
Q1J6G5_STRPF	GMP synthase (Glutamine-hydrolyzing) (EC 6.3.5.2) - *Streptococcus pyogenes* serotype M4 (strain MGAS10750)
Q1J6G9_STRPF	Signal recognition particle protein subunit FFH - *Streptococcus pyogenes* serotype M4 (strain MGAS10750)
Q1J6H7_STRPF	Citrate lyase beta chain / Citryl-CoA lyase subunit (EC 4.1.3.6) (EC 4.1.3.34) - *Streptococcus pyogenes* serotype M4 (strain MGAS10750)

Ac.No[a]	Protein name[b]
Q1J6J9_STRPF	DNA topoisomerase I (EC 5.99.1.2) - *Streptococcus pyogenes* serotype M4 (strain MGAS10750)
Q1J6U3_STRPF	NAD(P)H-dependent quinone reductase (EC 1.-.-.-) - *Streptococcus pyogenes* serotype M4 (strain MGAS10750)
Q1J6W8_STRPF	Phosphoglucosamine mutase (EC 5.4.2.10) - *Streptococcus pyogenes* serotype M4 (strain MGAS10750)
Q1J6X3_STRPF	Lipoate-protein ligase (EC 6.3.1.20) - *Streptococcus pyogenes* serotype M4 (strain MGAS10750)
Q1J719_STRPF	Branched-chain amino acid aminotransferase (EC 2.6.1.42) - *Streptococcus pyogenes* serotype M4 (strain MGAS10750)
Q1J790_STRPF	Periplasmic component of efflux system - *Streptococcus pyogenes* serotype M4 (strain MGAS10750)
Q1J7B8_STRPF	Translation Initiation Factor IF-3 - *Streptococcus pyogenes* serotype M4 (strain MGAS10750)
Q1J7C7_STRPF	Glycosyltransferase involved in cell wall biogenesis (EC 2.4.-.-) - *Streptococcus pyogenes* serotype M4 (strain MGAS10750)
Q1J7G0_STRPF	ATP synthase gamma chain (EC 3.6.3.14) - *Streptococcus pyogenes* serotype M4 (strain MGAS10750)
Q1J7I7_STRPF	Septation ring formation regulator EzrA - *Streptococcus pyogenes* serotype M4 (strain MGAS10750)
Q1J7N8_STRPF	Oligoendopeptidase F (EC 3.4.24.-) - *Streptococcus pyogenes* serotype M4 (strain MGAS10750)
Q1J7R7_STRPF	Signal recognition particle receptor FtsY - *Streptococcus pyogenes* serotype M4 (strain MGAS10750)
Q1J841_STRPF	Manganese-binding protein - *Streptococcus pyogenes* serotype M4 (strain MGAS10750)
Q1J842_STRPF	Iron-dependent repressor - *Streptococcus pyogenes* serotype M4 (strain MGAS10750)
Q1J844_STRPF	5'-methylthioadenosine nucleosidase / S-adenosylhomocysteine nucleosidase (EC 3.2.2.9) - *Streptococcus pyogenes* serotype M4 (strain MGAS10750)
Q1J7G1_STRPF	ATP synthase subunit alpha (EC 3.6.3.14) - *Streptococcus pyogenes* serotype M4 (strain MGAS10750)
Q1J7K8_STRPF	(R,R)-butanediol dehydrogenase / Acetoin dehydrogenase (EC 1.1.1.4) (EC 1.1.1.5) - *Streptococcus pyogenes* serotype M4 (strain MGAS10750)
Q1J7Q6_STRPF	HPr kinase/phosphorylase (EC 2.7.1.-) - *Streptococcus pyogenes* serotype M4 (strain MGAS10750)
Q1J885_STRPF	Thymidylate kinase (EC 2.7.4.9).- *Streptococcus pyogenes* serotype M4 (strain MGAS10750)
Q1J913_STRPF	LSU ribosomal protein L1E (L4P) - *Streptococcus pyogenes* serotype M4 (strain MGAS10750)
Q1J4L0_STRPF	Multiple sugar transport ATP-binding protein msmK - *Streptococcus pyogenes* serotype M4 (strain MGAS10750)
Q1J5S3_STRPF	GTP-binding protein TypA/BipA - *Streptococcus pyogenes* serotype M4 (strain MGAS10750)
Q1J6F7_STRPF	Formate--tetrahydrofolate ligase 1 (EC 6.3.4.3) - *Streptococcus pyogenes* serotype M4 (strain MGAS10750)
Q1J7D7_STRPF	RNA polymerase sigma factor SigA - *Streptococcus pyogenes* serotype M4 (strain MGAS10750)
Q1J6Z5_STRPF	dTDP-4-dehydrorhamnose 3,5-epimerase (EC 5.1.3.13) - *Streptococcus pyogenes* serotype M4 (strain MGAS10750)
Q1J822_STRPF	GTPase Era - *Streptococcus pyogenes* serotype M4 (strain MGAS10750)
Q1J8C3_STRPF	Transcription elongation factor GreA - *Streptococcus pyogenes* serotype M4 (strain MGAS10750)

Ac.No[a]	Protein name[b]
Q1J8I4_STRPF	Elongation factor G - *Streptococcus pyogenes* serotype M4 (strain MGAS10750)
Q1J8S9_STRPF	V-type sodium ATP synthase subunit K- *Streptococcus pyogenes* serotype M4 (strain MGAS10750)
Q1J8I5_STRPF	30S ribosomal protein S7 - *Streptococcus pyogenes* serotype M4 (strain MGAS10750)
Q1J723_STRPF	Dihydroorotase - *Streptococcus pyogenes* serotype M4 (strain MGAS10750)
Q1J8L9_STRPF	Multidrug/protein/lipid ABC transporter family, ATP-binding and permease protein - *Streptococcus pyogenes* serotype M4 (strain MGAS10750)
Q1J4K0_STRPF	Ribosomal protein L11 methyltransferase (EC 2.1.1.-) - *Streptococcus pyogenes* serotype M4 (strain MGAS10750)
Q1J849_STRPF	NAD-dependent oxidoreductase - *Streptococcus pyogenes* serotype M4 (strain MGAS10750)
Q1J6I5_STRPF	Transcriptional regulator, GntR family - *Streptococcus pyogenes* serotype M4 (strain MGAS10750)
Q1J4C0_STRPF	Formate--tetrahydrofolate ligase 2 - *Streptococcus pyogenes* serotype M4 (strain MGAS10750)

[a]Ac.No, access number of each protein in UniProt data bank

[b]Protein name, protein identification in UniProt data bank
