# Supplementary material for: Cellular Growth Arrest and Efflux Pumps Are Associated With Antibiotic Persisters in Streptococcus pyogenes Induced in Biofilm-Like Environments
Source: Front Microbiol. 2021 Sep 21;12:716628. doi: 10.3389/fmicb.2021.716628 (PMC8490960; doi:10.3389/fmicb.2021.716628)
Supplement: Supplementary Table 6 — Proteins identified in Streptococcus pyogenes strain 37–97 grown using low cell density. [file Data_Sheet_6.PDF]

Supplementary Table S6. Proteins identified in *Streptococcus pyogenes* strain 37-97 grown using low cell density.

Ac.No[a]	Protein name[b]
Q1J4F8_STRPF	Streptopain (EC 3.4.22.10) - *Streptococcus pyogenes* serotype M4 (strain MGAS10750)
Q1J5V7_STRPF	DNA-binding protein HU 1, chain A - *Streptococcus pyogenes* serotype M4 (strain MGAS10750)
Q1J6Q8_STRPF	2-amino-4-hydroxy-6-hydroxymethyldihydropteridine pyrophosphokinase - *Streptococcus pyogenes* serotype M4 (strain MGAS10750)
Q1J8I6_STRPF	30S ribosomal protein S12 - *Streptococcus pyogenes serotype M4* (strain MGAS10750)
Q1J459_STRPF	50S ribosomal protein L9 - *Streptococcus pyogenes* serotype M4 (strain MGAS10750)
Q1J8Z9_STRPF	30S ribosomal protein S8 - *Streptococcus pyogenes* serotype M4 (strain MGAS10750)
Q1J6D2_STRPF	Phosphate import ATP-binding protein pstB 1 (EC 3.6.3.27) - *Streptococcus pyogenes* serotype M4 (strain MGAS10750)
Q1J610_STRPF	Phosphocarrier protein HPr (Histidine-containing protein) - *Streptococcus pyogenes* serotype M4 (strain MGAS10750)
Q1J486_STRPF	Arginyl-tRNA synthetase (EC 6.1.1.19) - *Streptococcus pyogenes* serotype M4 (strain MGAS10750)
Q1J4B7_STRPF	Histidine ammonia-lyase (EC 4.3.1.3) - *Streptococcus pyogenes* serotype M4 (strain MGAS10750)
Q1J4Z3_STRPF	Deoxyribose-phosphate aldolase (EC 4.1.2.4) - *Streptococcus pyogenes* serotype M4 (strain MGAS10750)
Q1J522_STRPF	30S ribosomal protein S6 - *Streptococcus pyogenes* serotype M4 (strain MGAS10750)
Q1J594_STRPF	Serine-tRNA ligase (EC 6.1.1.11) - *Streptococcus pyogenes* serotype M4 (strain MGAS10750)
Q1J5D6_STRPF	N-acetylglucosamine-6-phosphate deacetylase (EC 3.5.1.25) - *Streptococcus pyogenes* serotype M4 (strain MGAS10750)
Q1J5D8_STRPF	Hypothetical membrane spanning protein - *Streptococcus pyogenes* serotype M4 (strain MGAS10750)
Q1J5G9_STRPF	Nicotinate phosphoribosyltransferase (EC 2.4.2.11) - *Streptococcus pyogenes* serotype M4(strain MGAS10750)
Q1J5H1_STRPF	Aminopeptidase C (EC 3.4.22.40) - *Streptococcus pyogenes* serotype M4 (strain MGAS10750)
Q1J5H5_STRPF	Cell cycle protein GpsB - *Streptococcus pyogenes* serotype M4 (strain MGAS10750)
Q1J5T6_STRPF	Isoleucine-tRNA ligase (EC 6.1.1.5) - *Streptococcus pyogenes* serotype M4 (strain MGAS10750)
Q1J663_STRPF	Glutamine transport ATP-binding protein glnQ - *Streptococcus pyogenes* serotype M4 (strain MGAS10750)
Q1J6R4_STRPF	Polysaccharide deacetylase - *Streptococcus pyogenes* serotype M4 (strain MGAS10750)
Q1J6X8_STRPF	Dihydrolipoamide acetyltransferase component of pyruvate dehydrogenase complex (EC 2.3.1.12).- *Streptococcus pyogenes* serotype M4 (strain MGAS10750)
Q1J7A9_STRPF	Glutathione reductase (EC 1.8.1.7) - *Streptococcus pyogenes* serotype M4 (strain MGAS10750)
Q1J7F1_STRPF	Phenylalanyl-tRNA synthetase beta subunit (EC 6.1.1.20) - *Streptococcus pyogenes* serotype M4 (strain MGAS10750)
Q1J7J0_STRPF	50S ribosomal protein L19 - *Streptococcus pyogenes* serotype M4 (strain MGAS10750)

Ac.No[a]	Protein name[b]
Q1J827_STRPF	Myosin-crossreactive antigen - *Streptococcus pyogenes* serotype M4 (strain MGAS10750)
Q1J835_STRPF	50S ribosomal protein L11 - *Streptococcus pyogenes* serotype M4 (strain MGAS10750)
Q1J8D7_STRPF	LemA protein - *Streptococcus pyogenes* serotype M4 (strain MGAS10750)
Q1J8E0_STRPF	Potassium uptake protein ktrA - *Streptococcus pyogenes* serotype M4 (strain MGAS10750)
Q1J8E6_STRPF	ABC transporter substrate-binding protein - *Streptococcus pyogenes* serotype M4 (strain MGAS10750)
Q1J8F1_STRPF	Ribosomal silencing factor RsfS - *Streptococcus pyogenes* serotype M4 (strain MGAS10750)
Q1J8I8_STRPF	Pur operon repressor - *Streptococcus pyogenes* serotype M4 (strain MGAS10750)
Q1J8Q8_STRPF	Leucine-tRNA ligase (EC 6.1.1.4) - *Streptococcus pyogenes* serotype M4 (strain MGAS10750)
Q1J8S0_STRPF	Adenylosuccinate synthetase (EC 6.3.4.4) - *Streptococcus pyogenes* serotype M4 (strain MGAS10750)
Q1J8S7_STRPF	V-type sodium ATP synthase subunit C - *Streptococcus pyogenes* serotype M4 (strain MGAS10750)
Q1J8T3_STRPF	Translation initiation inhibitor - *Streptococcus pyogenes* serotype M4 (strain MGAS10750)
Q1J8Z7_STRPF	50S ribosomal protein L18 - *Streptococcus pyogenes* serotype M4 (strain MGAS10750)
Q1J904_STRPF	50S ribosomal protein L14 - *Streptococcus pyogenes* serotype M4 (strain MGAS10750)
Q1J910_STRPF	30S ribosomal protein S19 - *Streptococcus pyogenes* serotype M4 (strain MGAS10750)
Q1J926_STRPF	Adenylosuccinate lyase (EC 4.3.2.2) - *Streptococcus pyogenes* serotype M4 (strain MGAS10750)
Q1J4D0_STRPF	10 kDa chaperonin - *Streptococcus pyogenes* serotype M4 (strain MGAS10750)
Q1J4P5_STRPF	50S ribosomal protein L13 - *Streptococcus pyogenes* serotype M4 (strain MGAS10750)
Q1J4X8_STRPF	Acid phosphatase (EC 3.1.3.2) - *Streptococcus pyogenes* serotype M4 (strain MGAS10750)
Q1J596_STRPF	PTS system, mannose-specific IID component (EC 2.7.1.69) - *Streptococcus pyogenes* serotype M4 (strain MGAS10750)
Q1J5A5_STRPF	Bis(5'-nucleosyl)-tetraphosphatase (Asymmetrical) (EC 3.6.1.17) - *Streptococcus pyogenes* serotype M4 (strain MGAS10750)
Q1J5N2_STRPF	Hypothetical cytosolic protein - *Streptococcus pyogenes* serotype M4 (strain MGAS10750)
Q1J7A0_STRPF	50S ribosomal protein L27 - *Streptococcus pyogenes* serotype M4 (strain MGAS10750)
Q1J7L0_STRPF	Cell division protein FtsX - *Streptococcus pyogenes* serotype M4 (strain MGAS10750)
Q1J7U2_STRPF	UDP-glucose 6-dehydrogenase (EC 1.1.1.22) - *Streptococcus pyogenes* serotype M4 (strain MGAS10750)
Q1J8D4_STRPF	Response regulator CsrR - *Streptococcus pyogenes* serotype M4 (strain MGAS10750)
Q1J8E3_STRPF	ABC transporter permease protein - *Streptococcus pyogenes* serotype M4 (strain MGAS10750)
Q1J8Z4_STRPF	50S ribosomal protein L15 - *Streptococcus pyogenes* serotype M4 (strain MGAS10750)
Q1J495_STRPF	Protein RecA - *Streptococcus pyogenes* serotype M4 (strain MGAS10750)

Ac.No[a]	Protein name[b]
Q1J4M6_STRPF	30S ribosomal protein S15 - *Streptococcus pyogenes* serotype M4 (strain MGAS10750)
Q1J4P6_STRPF	30S ribosomal protein S9 - *Streptococcus pyogenes* serotype M4 (strain MGAS10750)
Q1J562_STRPF	Universal stress protein family - *Streptococcus pyogenes* serotype M4 (strain MGAS10750)
Q1J6D8_STRPF	30S ribosomal protein S20 - *Streptococcus pyogenes* serotype M4 (strain MGAS10750)
Q1J6F9_STRPF	Anaerobic ribonucleoside-triphosphate reductase (EC 1.17.4.2) - *Streptococcus pyogenes* serotype M4 (strain MGAS10750)
Q1J6K6_STRPF	Uncharacterized protein - *Streptococcus pyogenes* serotype M4 (strain MGAS10750)
Q1J7E1_STRPF	Large-conductance mechanosensitive channel - *Streptococcus pyogenes* serotype M4 (strain MGAS10750)
Q1J834_STRPF	50S ribosomal protein L1 - *Streptococcus pyogenes* serotype M4 (strain MGAS10750)
Q1J8C6_STRPF	UDP-N-acetylmuramate--L-alanine ligase (EC 6.3.2.8) - *Streptococcus pyogenes* serotype M4 (strain MGAS10750)
Q1J907_STRPF	50S ribosomal protein L16 - *Streptococcus pyogenes* serotype M4 (strain MGAS10750)
Q1J909_STRPF	50S ribosomal protein L22 - *Streptococcus pyogenes* serotype M4 (strain MGAS10750)
Q1J6P7_STRPF	Class B acid phosphatase (EC 3.1.3.2) - *Streptococcus pyogenes* serotype M4 (strain MGAS10750)
Q1J6U0_STRPF	50S ribosomal protein L10 - *Streptococcus pyogenes* serotype M4 (strain MGAS10750)
Q1J6U5_STRPF	Succinate-semialdehyde dehydrogenase (NADP+) (EC 1.2.1.16) - *Streptococcus pyogenes* serotype M4 (strain MGAS10750)
Q1J8Y6_STRPF	50S ribosomal protein L17 - *Streptococcus pyogenes* serotype M4 (strain MGAS10750)
Q1J8Z8_STRPF	50S ribosomal protein L6 - *Streptococcus pyogenes* serotype M4 (strain MGAS10750)
Q1J912_STRPF	50S ribosomal protein L23 - *Streptococcus pyogenes* serotype M4 (strain MGAS10750)
Q1J479_STRPF	50S ribosomal protein L32 - *Streptococcus pyogenes* serotype M4 (strain MGAS10750)
Q1J917_STRPF	30S ribosomal protein S10 - *Streptococcus pyogenes* serotype M4 (strain MGAS10750)
Q1J4Z1_STRPF	Uridine phosphorylase (Fragment) - *Streptococcus pyogenes* serotype M4 (strain MGAS10750)
Q1J518_STRPF	Thioredoxin - *Streptococcus pyogenes* serotype M4 (strain MGAS10750)
Q1J5T0_STRPF	Cell division protein FtsZ - *Streptococcus pyogenes* serotype M4 (strain MGAS10750)
Q1J5M6_STRPF	Hypothetical cytosolic protein - *Streptococcus pyogenes* serotype M4 (strain MGAS10750)
Q1JNU5_STRPC	Transcriptional regulator, AraC family - *Streptococcus pyogenes* serotype M12 (strain MGAS9429)
Q1J6Z6_STRPF	Glucose-1-phosphate thymidylyltransferase - *Streptococcus pyogenes* serotype M4 (strain MGAS10750)
Q1J6T9_STRPF	50S ribosomal protein L7/L12 - *Streptococcus pyogenes* serotype M4 (strain MGAS10750)

[a]Ac.No, access number of each protein in UniProt data bank

[b]Protein name, protein identification in UniProt data bank
