# Supplementary material for: Cellular Growth Arrest and Efflux Pumps Are Associated With Antibiotic Persisters in Streptococcus pyogenes Induced in Biofilm-Like Environments
Source: Front Microbiol. 2021 Sep 21;12:716628. doi: 10.3389/fmicb.2021.716628 (PMC8490960; doi:10.3389/fmicb.2021.716628)
Supplement: Supplementary Table 7 — Proteins identified in Streptococcus pyogenes strain 37–97 grown in both biofilm-like environments and using low cell density. [file Data_Sheet_7.PDF]

Supplementary Table S7. Proteins identified in *Streptococcus pyogenes* strain 37-97 grown in both biofilm-like environments and using low cell density.

Ac.No[a]	Protein name[b]
Q1J8N2_STRPF	Glucose-6-phosphate isomerase - Streptococcus pyogenes serotype M4 (strain MGAS10750)
Q1J626_STRPF	S-adenosylmethionine synthase - *Streptococcus pyogenes* serotype M4 (strain MGAS10750)
Q1J8X0_STRPF	DNA-directed RNA polymerase subunit beta - *Streptococcus pyogenes* serotype M4 (strain MGAS10750)
Q1J4X3_STRPF	Fructose-bisphosphate aldolase (EC 4.1.2.13) - *Streptococcus pyogenes* serotype M4 (strain MGAS10750)
Q1J5Q4_STRPF	Arginine deiminase (EC 3.5.3.6) - *Streptococcus pyogenes* serotype M4 (strain MGAS10750)
Q1J694_STRPF	ATP-dependent 6-phosphofructokinase - *Streptococcus pyogenes* serotype M4 (strain MGAS10750)
Q1J578_STRPF	Chaperone protein DnaK - *Streptococcus pyogenes* serotype M4 (strain MGAS10750)
Q1J8I4_STRPF	Elongation factor G - *Streptococcus pyogenes* serotype M4 (strain MGAS10750)
Q1J7I5_STRPF	Enolase (EC 4.2.1.11) - *Streptococcus pyogenes* serotype M4 (strain MGAS10750)
Q1J441_STRPF	Inosine-5'-monophosphate dehydrogenase - *Streptococcus pyogenes* serotype M4 (strain MGAS10750)
Q1J6L1_STRPF	L-lactate dehydrogenase (EC 1.1.1.27) - *Streptococcus pyogenes* serotype M4 (strain MGAS10750)
Q1J7F9_STRPF	ATP synthase subunit beta - *Streptococcus pyogenes* serotype M4 (strain MGAS10750)
Q1J5Q6_STRPF	Ornithine carbamoyltransferase (EC 2.1.3.3) - *Streptococcus pyogenes* serotype M4 (strain MGAS10750)
Q1J7C3_STRPF	Peptidase T (EC 3.4.11.4) - *Streptococcus pyogenes* serotype M4 (strain MGAS10750)
Q1J4X9_STRPF	Phosphoglycerate kinase (EC 2.7.2.3) - *Streptococcus pyogenes* serotype M4 (strain MGAS10750)
Q1J5B8_STRPF	Copper-exporting ATPase - *Streptococcus pyogenes* serotype M4 (strain MGAS10750)
Q1J462_STRPF	30S ribosomal protein S4 - *Streptococcus pyogenes* serotype M4 (strain MGAS10750)
Q1J4C3_STRPF	Urocanate hydratase - *Streptococcus pyogenes* serotype M4 (strain MGAS10750)
Q1J4C5_STRPF	Peroxiredoxin reductase (NAD(P)H) / NADH oxidase H2O2-forming - *Streptococcus pyogenes* serotype M4 (strain MGAS10750)
Q1J4C6_STRPF	Peroxiredoxin (EC 1.11.1.15) - *Streptococcus pyogenes* serotype M4 (strain MGAS10750)
Q1J4D1_STRPF	60 kDa chaperonin - *Streptococcus pyogenes* serotype M4 (strain MGAS10750)
Q1J4X7_STRPF	Membrane protease protein family - *Streptococcus pyogenes* serotype M4 (strain MGAS10750)
Q1J4Y2_STRPF	Glutamine synthetase (EC 6.3.1.2) - *Streptococcus pyogenes* serotype M4 (strain MGAS10750)
Q1J539_STRPF	Mannose-6-phosphate isomerase (EC 5.3.1.8) - *Streptococcus pyogenes* serotype M4 (strain MGAS10750)
Q1J569_STRPF	Glutamyl-tRNA (Gln) amidotransferase subunit A - *Streptococcus pyogenes* serotype M4 (strain MGAS10750)

Ac.No[a]	Protein name[b]
Q1J588_STRPF	3-oxoacyl-[acyl-carrier-protein] synthase 2 - *Streptococcus pyogenes* serotype M4 (strain MGAS10750)
Q1J5B4_STRPF	Translation initiation factor IF-2 - *Streptococcus pyogenes* serotype M4 (strain MGAS10750)
Q1J5E0_STRPF	Glycine--tRNA ligase alpha subunit (EC 6.1.1.14) - *Streptococcus pyogenes* serotype M4 (strain MGAS10750)
Q1J5H7_STRPF	Mid-cell-anchored protein Z - *Streptococcus pyogenes* serotype M4 (strain MGAS10750)
Q1J5J2_STRPF	Serine/threonine protein kinase - *Streptococcus pyogenes* serotype M4 (strain MGAS10750)
Q1J5J8_STRPF	Cysteine synthase (EC 2.5.1.47) - *Streptococcus pyogenes* serotype M4 (strain MGAS10750)
Q1J5Q7_STRPF	Arginine/ornithine antiporter - *Streptococcus pyogenes* serotype M4 (strain MGAS10750)
Q1J5Q9_STRPF	Carbamate kinase - *Streptococcus pyogenes* serotype M4 (strain MGAS10750)
Q1J5S1_STRPF	Glucokinase - *Streptococcus pyogenes* serotype M4 (strain MGAS10750)
Q1J5T9_STRPF	ATP-dependent endopeptidase clp ATP-binding subunit clpE - *Streptococcus pyogenes* serotype M4 (strain MGAS10750)
Q1J612_STRPF	NADP-dependent glyceraldehyde-3-phosphate dehydrogenase - *Streptococcus pyogenes* serotype M4 (strain MGAS10750)
Q1J664_STRPF	Glutamine-binding protein / Glutamine transport system permease protein glnP - *Streptococcus pyogenes* serotype M4 (strain MGAS10750)
Q1J686_STRPF	Alpha-1,4 glucan phosphorylase (EC 2.4.1.1) - *Streptococcus pyogenes* serotype M4 (strain MGAS10750)
Q1J6A8_STRPF	Uncharacterized protein- *Streptococcus pyogenes* serotype M4 (strain MGAS10750)
Q1J6D5_STRPF	Aminopeptidase (EC 3.4.11.-) - *Streptococcus pyogenes* serotype M4 (strain MGAS10750)
Q1J6L0_STRPF	DNA gyrase subunit A (EC 5.99.1.3) - *Streptococcus pyogenes* serotype M4 (strain MGAS10750)
Q1J6L2_STRPF	NADH oxidase H2O-forming (EC 1.6.-.-) - *Streptococcus pyogenes* serotype M4 (strain MGAS10750)
Q1J6N2_STRPF	Phosphate acetyltransferase (EC 2.3.1.8) - *Streptococcus pyogenes* serotype M4 (strain MGAS10750)
Q1J6V1_STRPF	PTS system, mannose/fructose family IIB component - *Streptococcus pyogenes* serotype M4 (strain MGAS10750)
Q1J733_STRPF	Purine nucleoside phosphorylase DeoD-type (EC 2.4.2.1) - *Streptococcus pyogenes* serotype M4 (strain MGAS10750)
Q1J738_STRPF	ATP-dependent endopeptidase clp ATP-binding subunit clpL - *Streptococcus pyogenes* serotype M4 (strain MGAS10750)
Q1J7K1_STRPF	Dipeptidase A (EC 3.4.13.-) - *Streptococcus pyogenes* serotype M4 (strain MGAS10750)
Q1J7K5_STRPF	Asparagine--tRNA ligase (EC 6.1.1.22) - *Streptococcus pyogenes* serotype M4 (strain MGAS10750)
Q1J7K9_STRPF	Hydroxyacylglutathione hydrolase (EC 3.1.2.6) - *Streptococcus pyogenes* serotype M4 (strain MGAS10750)
Q1J7M6_STRPF	Calcium-transporting ATPase (EC 3.6.3.8) - *Streptococcus pyogenes* serotype M4 (strain MGAS10750)
Q1J7N3_STRPF	Triosephosphate isomerase (EC 5.3.1.1) - *Streptococcus pyogenes* serotype M4 (strain MGAS10750)
Q1J7N4_STRPF	Elongation factor Tu - *Streptococcus pyogenes* serotype M4 (strain MGAS10750)

Ac.No[a]	Protein name[b]
Q1J832_STRPF	Ribosome-recycling factor - *Streptococcus pyogenes* serotype M4 (strain MGAS10750)
Q1J873_STRPF	L-lactate oxidase (EC 1.13.12.-) - *Streptococcus pyogenes* serotype M4 (strain MGAS10750)
Q1J898_STRPF	Probable manganese-dependent inorganic pyrophosphatase (EC 3.6.1.1) - *Streptococcus pyogenes* serotype M4 (strain MGAS10750)
Q1J8I3_STRPF	Glyceraldehyde-3-phosphate dehydrogenase - *Streptococcus pyogenes* serotype M4 (strain MGAS10750)
Q1J8L4_STRPF	Uncharacterized protein - *Streptococcus pyogenes* serotype M4 (strain MGAS10750)
Q1J8P6_STRPF	DNA polymerase I (EC 2.7.7.7) - *Streptococcus pyogenes* serotype M4 (strain MGAS10750)
Q1J8S4_STRPF	V-type ATP synthase beta chain - *Streptococcus pyogenes* serotype M4 (strain MGAS10750)
Q1J8S5_STRPF	V-type ATP synthase alpha chain (EC 3.6.3.15) - *Streptococcus pyogenes* serotype M4 (strain MGAS10750)
Q1J8W0_STRPF	Acetate kinase (EC 2.7.2.1) - *Streptococcus pyogenes* serotype M4 (strain MGAS10750)
Q1J8Y8_STRPF	30S ribosomal protein S11 - *Streptococcus pyogenes* serotype M4 (strain MGAS10750)
Q1J8Y9_STRPF	30S ribosomal protein S13 - *Streptococcus pyogenes* serotype M4 (strain MGAS10750)
Q1J911_STRPF	50S ribosomal protein L2 - *Streptococcus pyogenes* serotype M4 (strain MGAS10750)
Q1J919_STRPF	Alcohol dehydrogenase (EC 1.1.1.1) - *Streptococcus pyogenes* serotype M4 (strain MGAS10750)
Q1J508_STRPF	Formate acetyltransferase (EC 2.3.1.54) - *Streptococcus pyogenes* serotype M4 (strain MGAS10750)
Q1J948_STRPF	ATP-dependent zinc metalloprotease FtsH (EC 3.4.24.-) - *Streptococcus pyogenes* serotype M4 (strain MGAS10750)
Q1J597_STRPF	PTS system, mannose-specific IIC component (EC 2.7.1.69) - *Streptococcus pyogenes* serotype M4 (strain MGAS10750)
Q1J611_STRPF	Phosphoenolpyruvate-protein phosphotransferase (EC 2.7.3.9) - *Streptococcus pyogenes* serotype M4 (strain MGAS10750)
Q1J6B2_STRPF	General stress protein, Gls24 family - *Streptococcus pyogenes* serotype M4 (strain MGAS10750)
Q1J6X6_STRPF	Dihydrolipoyl dehydrogenase (EC 1.8.1.4) - *Streptococcus pyogenes* serotype M4 (strain MGAS10750)
Q1J715_STRPF	SSU ribosomal protein S1P - *Streptococcus pyogenes* serotype M4 (strain MGAS10750)
Q1J7A2_STRPF	50S ribosomal protein L21 - *Streptococcus pyogenes* serotype M4 (strain MGAS10750)
Q1J7B6_STRPF	50S ribosomal protein L20 - *Streptococcus pyogenes* serotype M4 (strain MGAS10750)
Q1J7Y1_STRPF	Pyrrolidone-carboxylate peptidase - *Streptococcus pyogenes* serotype M4 (strain MGAS10750)
Q1J8Z3_STRPF	Protein translocase subunit SecY - *Streptococcus pyogenes* serotype M4 (strain MGAS10750)
Q1J8Z6_STRPF	30S ribosomal protein S5 - *Streptococcus pyogenes* serotype M4 (strain MGAS10750)
Q1J901_STRPF	50S ribosomal protein L5 - *Streptococcus pyogenes* serotype M4 (strain MGAS10750)
Q1J916_STRPF	LSU ribosomal protein L3P - *Streptococcus pyogenes* serotype M4 (strain MGAS10750)

Ac.No[a]	Protein name[b]
Q1J4B3_STRPF	Elongation factor Ts - *Streptococcus pyogenes* serotype M4 (strain MGAS10750)
Q1J4B4_STRPF	30S ribosomal protein S2 - *Streptococcus pyogenes* serotype M4 (strain MGAS10750)
Q1J4G1_STRPF	Foldase protein PrsA (EC 5.2.1.8) - *Streptococcus pyogenes* serotype M4 (strain MGAS10750)
Q1J4N2_STRPF	Polyribonucleotide nucleotidyltransferase (EC 2.7.7.8) - *Streptococcus pyogenes* serotype M4 (strain MGAS10750)
Q1J586_STRPF	Malonyl CoA-acyl carrier protein transacylase (EC 2.3.1.39 - *Streptococcus pyogenes* serotype M4 (strain MGAS10750)
Q1J587_STRPF	3-oxoacyl-reductase (EC 1.1.1.100) - *Streptococcus pyogenes* serotype M4 (strain MGAS10750)
Q1J593_STRPF	Acetyl-coenzyme A carboxylase carboxyl transferase subunit alpha - *Streptococcus pyogenes* serotype M4 (strain MGAS10750)
Q1J5K2_STRPF	Ribosome hibernation promoting factor - *Streptococcus pyogenes* serotype M4 (strain MGAS10750)
Q1J5W1_STRPF	2,3-bisphosphoglycerate-dependent phosphoglycerate mutase - *Streptococcus pyogenes* serotype M4 (strain MGAS10750)
Q1J691_STRPF	ABC transporter ATP-binding protein - *Streptococcus pyogenes* serotype M4 (strain MGAS10750)
Q1J695_STRPF	Pyruvate kinase (EC 2.7.1.40) - *Streptococcus pyogenes* serotype M4 (strain MGAS10750)
Q1J6Y0_STRPF	Pyruvate dehydrogenase E1 component alpha subunit (EC 1.2.4.1) - *Streptococcus pyogenes* serotype M4 (strain MGAS10750)
Q1J6Z4_STRPF	dTDP-glucose 4,6-dehydratase (EC 4.2.1.46) - *Streptococcus pyogenes* serotype M4 (strain MGAS10750)
Q1J702_STRPF	Adenine phosphoribosyltransferase (EC 2.4.2.7) - *Streptococcus pyogenes* serotype M4 (strain MGAS10750)
Q1J736_STRPF	Phosphopentomutase (EC 5.4.2.7) - *Streptococcus pyogenes* serotype M4 (strain MGAS10750)
Q1J7H4_STRPF	Streptolysin S export ATP-binding protein SagG - *Streptococcus pyogenes* serotype M4 (strain MGAS10750)
Q1J7K4_STRPF	Nucleotide-binding protein MGAS10750_Spy0557 - *Streptococcus pyogenes* serotype M4 (strain MGAS10750)
Q1J7X4_STRPF	Catabolite control protein A - *Streptococcus pyogenes* serotype M4 (strain MGAS10750)
Q1J943_STRPF	Ribose-phosphate pyrophosphokinase (EC 2.7.6.1) - *Streptococcus pyogenes* serotype M4 (strain MGAS10750)
Q1J4C9_STRPF	Negative regulator of genetic competence clpC/mecB - *Streptococcus pyogenes* serotype M4 (strain MGAS10750)
Q1J5R9_STRPF	Non-specific DNA-binding protein Dps / Iron-binding ferritin-like antioxidant protein / Ferroxidase - *Streptococcus pyogenes* serotype M4 (strain MGAS10750)
Q1JKY8_STRPF	Cell division protein FtsZ - *Streptococcus pyogenes* serotype M4 (strain MGAS10750)
Q1J6B0_STRPF	General stress protein, Gls24 family - *Streptococcus pyogenes* serotype M4 (strain MGAS10750)
Q1J888_STRPF	Uracil phosphoribosyltransferase (EC 2.4.2.9) - *Streptococcus pyogenes* serotype M4 (strain MGAS10750)
Q1J908_STRPF	30S ribosomal protein S3 - *Streptococcus pyogenes* serotype M4 (strain MGAS10750)

Ac.No[a]	Protein name[b]
Q1J959_STRPF	DNA polymerase III subunit beta (EC 2.7.7.7) - *Streptococcus pyogenes* serotype M4 (strain MGAS10750)
Q1J6S4_STRPF	Lantibiotic transport ATP-binding protein - *Streptococcus pyogenes* serotype M4 (strain MGAS10750)
Q1J6U9_STRPF	PTS system, mannose/fructose family IID component - *Streptococcus pyogenes* serotype M4 (strain MGAS10750)
Q1J7G3_STRPF	ATP synthase subunit b - *Streptococcus pyogenes* serotype M4 (strain MGAS10750)
Q1J5F1_STRPF	Transketolase (EC 2.2.1.1) - *Streptococcus pyogenes* serotype M4 (strain MGAS10750)
Q1J6D3_STRPF	Phosphate-specific transport system accessory protein PhoU - *Streptococcus pyogenes* serotype M4 (strain MGAS10750)
Q1J5U2_STRPF	Arginine transport ATP-binding protein artP - *Streptococcus pyogenes* serotype M4 (strain MGAS10750)
Q1J6X9_STRPF	Pyruvate dehydrogenase E1 component beta subunit - *Streptococcus pyogenes* serotype M4 (strain MGAS10750)
Q1J5Y1_STRPF	Superoxide dismutase (EC 1.15.1.1) - *Streptococcus pyogenes* serotype M4 (strain MGAS10750)
Q1J4L9_STRPF	Proline--tRNA ligase (EC 6.1.1.15) - *Streptococcus pyogenes* serotype M4 (strain MGAS10750)
Q1J530_STRPF	Elongation factor P - *Streptococcus pyogenes* serotype M4 (strain MGAS10750)
Q1J543_STRPF	Protein translocase subunit SecA - *Streptococcus pyogenes* serotype M4 (strain MGAS10750)
Q1J561_STRPF	Hydrolase - *Streptococcus pyogenes* serotype M4 (strain MGAS10750)
Q1J864_STRPF	Methionine--tRNA ligase (EC 6.1.1.10) - *Streptococcus pyogenes* serotype M4 (strain MGAS10750)
Q1J8G5_STRPF	Oligopeptide-binding protein oppA - *Streptococcus pyogenes* serotype M4 (strain MGAS10750)
Q1J8R9_STRPF	Nucleoside-binding protein - *Streptococcus pyogenes* serotype M4 (strain MGAS10750)
Q1J585_STRPF	Enoyl-reductase (NADH) (EC 1.3.1.9) - *Streptococcus pyogenes* serotype M4 (strain MGAS10750)
Q1J598_STRPF	PTS system, mannose-specific IIAB component (EC 2.7.1.69) - *Streptococcus pyogenes* serotype M4 (strain MGAS10750)
Q1J673_STRPF	Maltose/maltodextrin-binding protein - *Streptococcus pyogenes* serotype M4 (strain MGAS10750)
Q1J815_STRPF	30S ribosomal protein S7 - *Streptococcus pyogenes* serotype M4 (strain MGAS10750)
Q1J8M2_STRPF	Glycerol-3-phosphate dehydrogenase [NAD(P)+] (EC 1.1.1.94) - *Streptococcus pyogenes* serotype M4 (strain MGAS10750)
Q1J8Y7_STRPF	DNA-directed RNA polymerase subunit alpha (EC 2.7.7.6) - *Streptococcus pyogenes* serotype M4 (strain MGAS10750)
Q1J6Y4_STRPF	Ribonuclease J - *Streptococcus pyogenes* serotype M4 (strain MGAS10750)
Q1J8X1_STRPF	DNA-directed RNA polymerase subunit beta - *Streptococcus pyogenes* serotype M4 (strain MGAS10750)
Q1J6U2_STRPF	Xaa-His dipeptidase - *Streptococcus pyogenes* serotype M4 (strain MGAS10750)

[a]Ac.No, access number of each protein in UniProt data bank

[b]Protein name, protein identification in UniProt data bank
